# In-situ nanospectroscopic imaging of plasmon-induced two-dimensional [4+4]-cycloaddition polymerization on Au(111)

**DOI:** 10.1038/s41467-021-24856-5

**Published:** 2021-07-27

**Authors:** Feng Shao, Wei Wang, Weimin Yang, Zhilin Yang, Yao Zhang, Jinggang Lan, A. Dieter Schlüter, Renato Zenobi

**Affiliations:** 1 Department of Physics and Astronomy, National Graphene Institute, University of Manchester, Manchester, UK; 2 Department of Chemistry and Applied Biosciences, ETH Zurich, Zurich, Switzerland; 3 Shanghai Key Laboratory of Green Chemistry and Chemical Processes, School of Chemistry and Molecular Engineering, Chang-Kung Chuang Institute, East China Normal University, Shanghai, People’s Republic of China; 4 Department of Physics, Collaborative Innovation Center for Optoelectronic Semiconductors and Efficient Devices, Jiujiang Research Institute, Xiamen University, Xiamen, Fujian People’s Republic of China; 5 Hefei National Laboratory for Physical Sciences at the Microscale and Synergetic Innovation Center of Quantum Information & Quantum Physics, University of Science and Technology of China, Hefei, People’s Republic of China; 6 Department of Chemistry, University of Zurich, Zurich, Switzerland; 7 Department of Materials, Polymer Chemistry, ETH Zurich, Zurich, Switzerland

**Keywords:** Imaging studies, Polymerization mechanisms, Raman spectroscopy, Two-dimensional materials

## Abstract

Plasmon-induced chemical reactions (PICRs) have recently become promising approaches for highly efficient light-chemical energy conversion. However, an in-depth understanding of their mechanisms at the nanoscale still remains challenging. Here, we present an in-situ investigation by tip-enhanced Raman spectroscopy (TERS) imaging of the plasmon-induced [4+4]-cycloaddition polymerization within anthracene-based monomer monolayers physisorbed on Au(111), and complement the experimental results with density functional theory (DFT) calculations. This two-dimensional (2D) polymerization can be flexibly triggered and manipulated by the hot carriers, and be monitored simultaneously by TERS in real time and space. TERS imaging provides direct evidence for covalent bond formation with ca. 3.7 nm spatial resolution under ambient conditions. Combined with DFT calculations, the TERS results demonstrate that the lateral polymerization on Au(111) occurs by a hot electron tunneling mechanism, and crosslinks form via a self-stimulating growth mechanism. We show that TERS is promising to be plasmon-induced nanolithography for organic 2D materials.

## Abstract (web summary)

Here, the authors use tip-enhanced Raman spectroscopy for in-situ investigation of plasmon-induced [4+4]-cycloaddition polymerization on Au(111). They find that this occurs by a hot electron tunneling mechanism, while crosslinks form via a self-stimulating growth mechanism.

## Introduction

Localized surface plasmon (LSP) resonances of metallic nanostructures can harvest and confine light near a metal surface via photon–electron interactions^1–3^, in the meantime the generated and enhanced local electric field enables a variety of applications, including near-field optical spectroscopies^4,5^, ultrasensitive biosensing^6^, photothermal therapy^7^, and plasmon-induced chemical reactions (PICRs)^8,9^. As the driving force for PICRs, LSPs lead to confined electromagnetic fields, charge transfer, local heat generation, as well as hot carrier excitation that facilitate multitudinous photoelectrocatalytic reactions^10–13^. Remarkably, hot carriers (hot electron-hole pairs), generated upon non-radiative decay of LSPs, are known to activate bond formation/dissociation which is challenging with conventional photocatalysts^10^, e.g., hydrogen dissociation^14^, ethylene epoxidation^15^, water splitting^16^, Suzuki coupling^17^, and ammonia synthesis/decomposition^11,18^. Understanding the interaction between incident light, hot carriers, and target molecules during PICRs at the nanoscale will help to recognize the reaction mechanisms and promote the chemical transformation of the plasmon-mediated photocatalysis, which in turn should provide insights into how to rationally design efficient plasmonic catalysts.

Conventional imaging techniques, such as super-resolution fluorescence microscopy^19,20^, dark-field optical spectroscopy^21,22^, and surface-enhanced Raman spectroscopy (SERS)^3,23^, have allowed for identifying the reactive sites, monitoring the reagent diffusion, and revealing the particle-to-particle variability of PICRs. However, these methods either lack the nanoscale spatial resolution to elucidate the site-activity relationship, or require fluorescent labels, which complicates the interpretation of the results^24^. Alternatively, in-situ environmental transmission electron microscopy (TEM) has been used for label-free monitoring of photocatalytic dehydrogenation with sub-2 nm spatial resolution, although there is no chemical information available during the photochemical processes^10^. Tip-enhanced Raman spectroscopy (TERS) bridges this gap because it simultaneously provides (sub)nanometer spatial resolution and Raman fingerprint information in a label-free fashion^25–28^. The most widely investigated PICRs by TERS include the dimerization of *p*-aminothiophenol or *p*-nitrothiophenol^24,29,30^, polymerization of dibenzo(1,2)dithiine-3,8-diamine^31^, dehydrogenation of 2,13-bis(aldehyde)-[7]thiaheterohelicene^32^, crosslinking of benzenemethanethiol^33^, deprotonation of 2-pyridinethiol^34^, and hydrogenation of chloronitrobenzenethiol^35^, which mainly rely on the thiol-anchored self-assembled monolayers (MLs) as the reaction models. However, these chemisorbed molecular MLs are untransferable, and LSPs from TERS hotspots cannot locally control such chemical systems to form covalent 2D materials.

[4+4]-Photocycloaddition between neighboring anthracene pairs with a face-to-face stacked packing can afford the controlled lateral polymerization of suitable monomers both in single crystals and in Langmuir–Blodgett (LB) MLs on the air/water interface, resulting in the formation of two-dimensional polymers (2DPs)^36,37^. 2DPs are expected to play important roles in next-generation organic 2D materials for catalysis, sensing, optoelectronics, and separating techniques^38–43^. The mechanism of 2D polymerization has been studied in layered single crystals. Most cases follow the so-called self-impeding growth, although there appears to be one case, in which self-stimulating growth prevails^44^. Upon UV-irradiation, photopolymerization leading to 2D polymers has also been realized on a Au(111) substrate^45^. Preparation of amphiphilic monomer/polymer MLs at an air/water interface allows for convenient transfer onto Au(111) by the Schäfer technique^46^. For MLs of structurally closely related monomers^47–49^, earlier work has shown that the excimer fluorescence of anthracene pairs contained in the monomer lattice before and after transfer remained unchanged. Additionally, for structural analysis of molecular MLs, our previous TERS imaging provided direct evidence for the monomers’ functional groups, newly formed chemical bonds, molecular orientation, intermolecular interaction, conversion of polymerization, and nanodefective domains^38,39,47^.

**Fig. 1:**
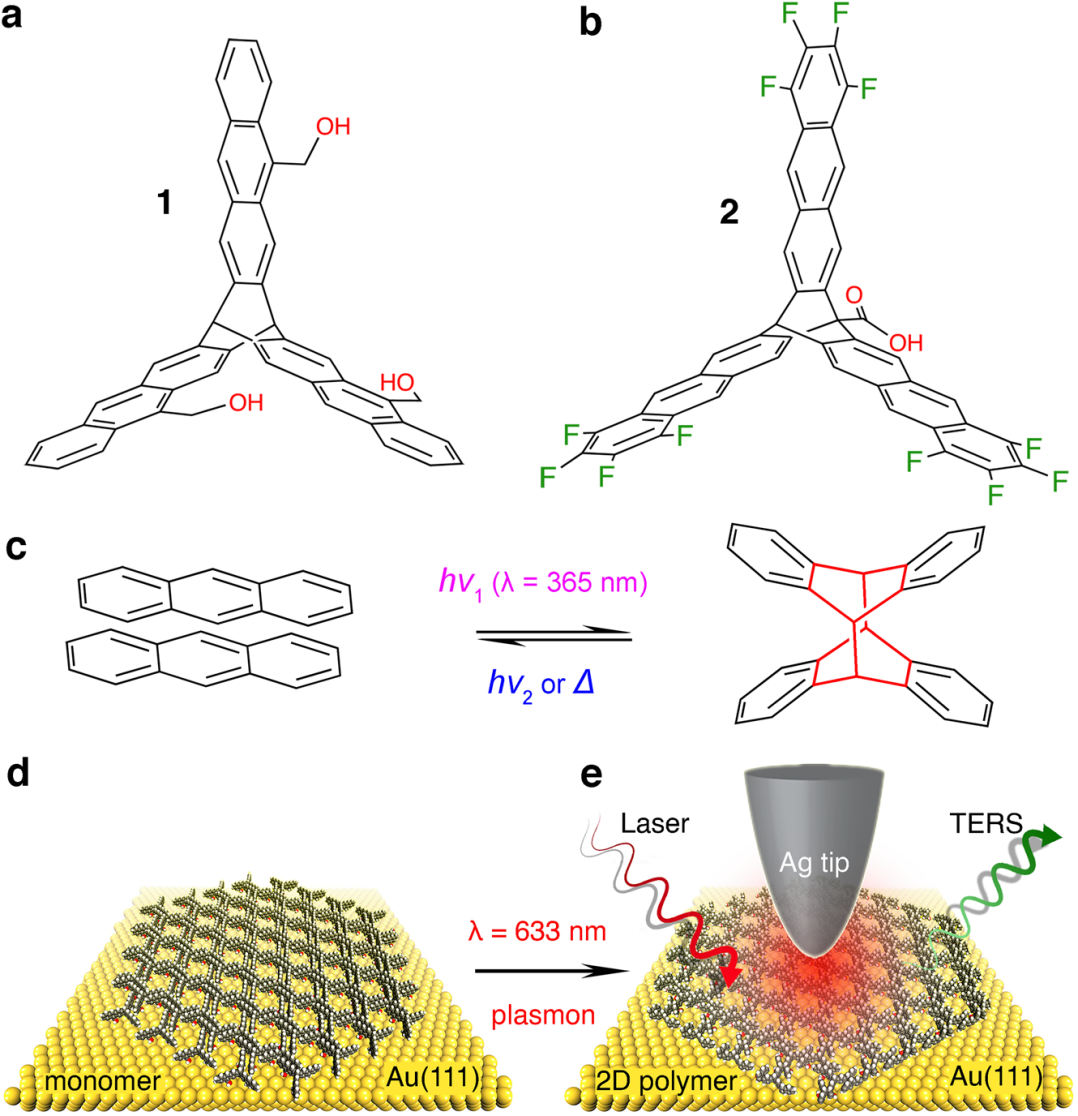
UV-driven and plasmon-induced [4+4]-cycloaddition. **a**, **b** Chemical structure of monomers **1** and **2**, respectively. **c** The reversible photochemical reaction between two anthracenes. The [4+4]-photocycloaddition reaction can be triggered by UV laser irradiation (365 nm, ca. 3.40 eV) and can also be reversed by irradiation at shorter wavelengths or thermally. **d** Schematic illustration of the monomer ML on Au(111). **e** Schematic illustration of the TERS imaging for the plasmon-induced 2DP ML under visible laser irradiation (633 nm, ca. 1.96 eV). *λ*, wavelength. *hν* laser light. *∆* heating.

**Fig. 2:**
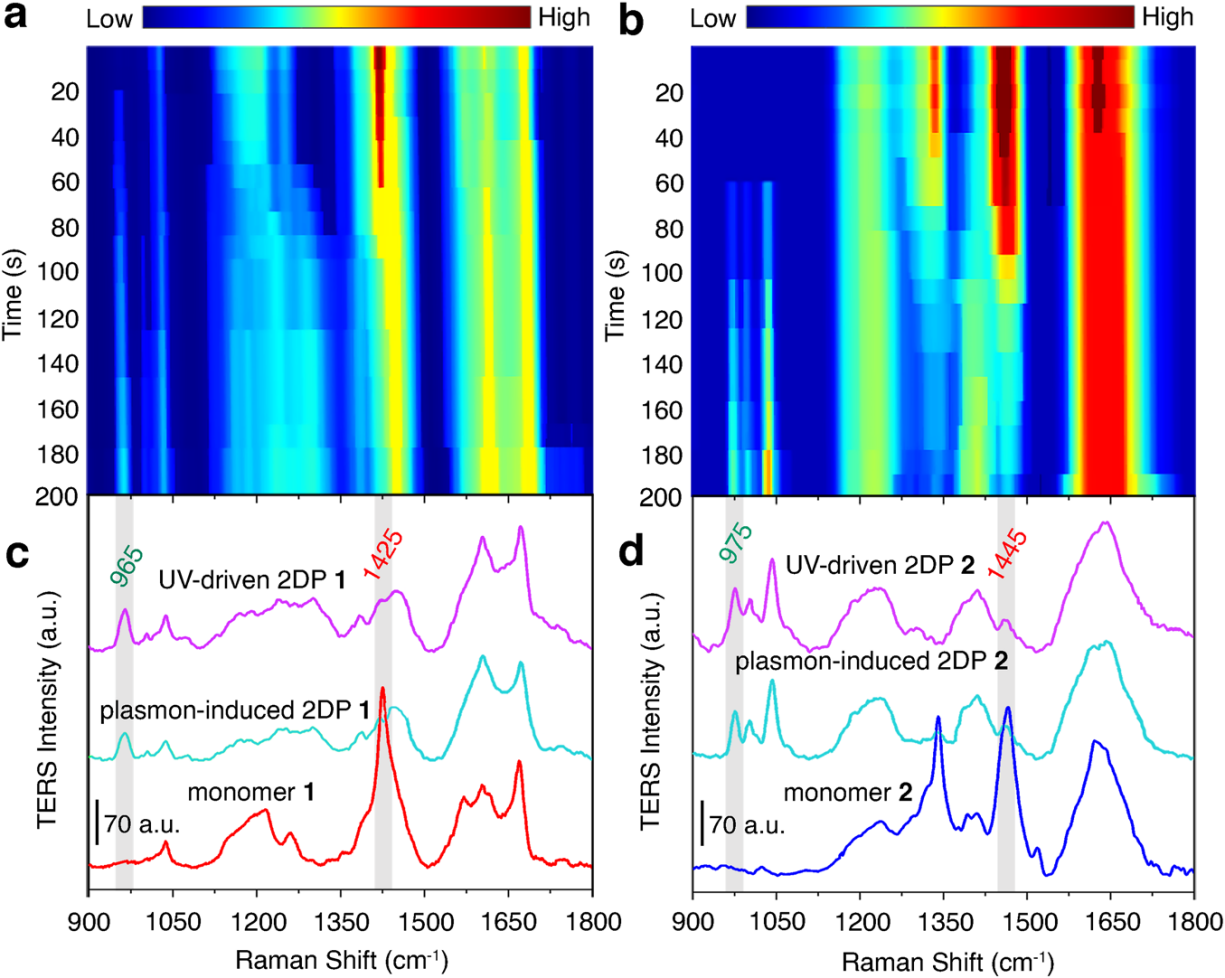
In-situ TERS study of the plasmon-induced 2D polymerization. **a**, **b** Time-dependent TER spectral evolution of monomer **a** **1** and **b** **2** MLs in the STM-TERS nanogap (0.1 V, 0.1 nA) under laser excitation (633 nm, 0.3 mW). Serial TER spectra were acquired with an integration time of 10 s. **c**, **d** Typical TER spectra of the corresponding monomer and 2DP MLs under different conditions. a.u. arbitrary units.

Here, as a proof-of-concept, we study a plasmon-induced [4+4]-cycloaddition in-situ, using 2D arrays of anthracene-based monomers deposited on Au(111) using TERS imaging and DFT calculations. We also compare these data with results from UV-driven polymerization. Amphiphilic monomers **1** and **2** were designed to have three anthracene-based blades connected through a triptycene core (Fig. 1a, b and Supplementary Fig. 1). They exhibit an antiparallel packing of the anthracene blades that are perfectly positioned for [4+4]-photocycloaddition to form 2DPs^46,47^. The three hydroxyl groups in **1** and the carboxylic acid group in **2** are expected to help the monomers lay down at the air/water interface, while the different substituent groups and locations in **1** (the hydroxymethyl groups in the middle benzene rings, Figs. 1a and 2) (the tetrafluorine atoms in the end benzene rings, Fig. 1b) are chosen to study substituent-related and spatial effects during plasmon-induced polymerization^46,50^. Generally, an anthracene-based [4+4]-photocycloaddition is triggered by a UV laser (e.g., 365 nm, ca. 3.40 eV) and can be reversed by irradiation at shorter wavelengths (e.g., 254 nm, ca. 4.88 eV) or thermally (e.g., 180–200 °C, Fig. 1c)^37,51^. However, we found that the 2D polymerization reaction can also be activated in the Ag tip/Au substrate nanogap under visible laser excitation (633 nm, ca. 1.96 eV, Fig. 1d, e). Both the monomer and 2DP MLs are physisorbed and only show weak interaction with the Au(111) surface, such that this becomes a useful model system to study the reaction mechanisms. The TERS configuration allows us to monitor the photocatalytic reaction in real-time and space. Accordingly, TERS discloses the excitation mechanism of the hot carrier transfer and visualizes the growth mechanism of the in-plane crosslinking at the nanoscale. The reactivity of the monomers can be affected by substituents and spatial effects caused by their structural differences. DFT calculations suggest that the plasmon-induced 2D polymerization occurs on Au(111) when the plasmon energy matches the lowest-unoccupied molecular orbital (LUMO) of the monomer MLs, and that the hot electrons can be inelastically transferred from Au(111) to the monomers although it only has a weak interaction with the metal surface.

## Results

### Plasmon-induced [4+4]-cycloaddition polymerization in the monomer MLs

Monomers **1** and **2** were designed and synthesized according to our previous work (Fig. 1a, b)^46,47^, which also described the preparation of corresponding monomer/2DP MLs and their packing behavior at the air/water interface. Neither monomer absorbs at the wavelength of the TERS excitation laser (Supplementary Fig. 2). The surface adsorption energy of monomer **1** and **2** MLs on Au(111) (Supplementary Fig. 3) was calculated to be ca. −0.66 eV and −0.49 eV for each anthracene blade, respectively, indicating that only a physisorption interaction between the MLs and Au(111) exists (Supplementary Fig. 4). According to the Woodward–Hoffmann rules, anthracene-based [4π_s_+4π_s_]-cycloaddition is thermally forbidden but photochemically allowed, involving the triplet excited state and the π → π* transition^52^. The calculated reaction barrier for anthracene dimerization was estimated to be ca. 2.39 eV^53^, an energy level that is higher than the energy from the TERS excitation laser (633 nm, ca. 1.96 eV). To obtain reference 2DPs, the monomer MLs were first irradiated on the LB with a conventional UV lamp (365 nm, ca. 3.40 eV) and then transferred onto substrates by the Schäfer technique for further characterization (Supplementary Figs. 5–12 and Supplementary Note 1). Based on the DFT-optimized models, π–π stacking structures with a distance of ca. 3.74 Å (**1**) and 3.68 Å (**2**) are expected to form in a densely packed array of the monomer MLs, respectively (Supplementary Fig. 5). Uniform hexagonal units with a size of ca. 22.34 Å (2DP **1**) and 22.36 Å (2DP **2**) are expected in the 2DP MLs after UV light irradiation, respectively (Supplementary Fig. 6). Particularly, previous high-resolution atomic force microscope (AFM) imaging in ultrahigh vacuum illustrated the high uniformity and crystallinity of 2DP **2** MLs^54^, indicating that the [4+4]-cycloaddition conversion is feasible for creating advanced 2D materials. Moreover, taking surface selection rules and intermolecular π–π stacking interactions into consideration (Supplementary Figs. 13 and 14), TERS DFT calculations were carried out to elucidate the molecular orientation of all the MLs. This confirms that these MLs lie flat on Au(111) with a face-to-face stacked packing^46^.

In our gap-mode TERS configuration, the Ag tip (curvature radius ca. 50 nm, Supplementary Fig. 15) plays a dual role: (i) as a single-particle catalyst enabling precise spatial control of plasmon-mediated polymerization thereby forming a covalently bonded 2D network; and (ii) as Raman signal booster enabling in-situ monitoring of photocatalytic processes for exploring the reaction mechanism (see Supplementary Note 2 for more details). According to finite-difference time-domain (FDTD) theoretical calculations (Supplementary Figs. 16–19)^55^, the local electric field is dramatically enhanced under 633 nm laser excitation with different incident directions. These enhanced locations are known as “hotspots”. Notably, due to the frequency matching and electromagnetic coupling, the incident laser can accordingly excite almost the strongest plasmon resonance in our hotspots (Supplementary Figs. 16–19). Raman signal enhancement (*G* ∝ log(|*E*_0_/*E*|^4^)) within these hotspots is calculated to be more than 10^8^, thereby facilitating the ultrasensitive monitoring of the 2D polymerization reaction. Based on our calculation results: (i) the mean molecular areas (MMA) of the monomer monolayers have been estimated to be ca. 153 Å^2^ (**1**) and 158 Å^2^ (**2**) per molecule (Supplementary Fig. 5), respectively, and (ii) the strongest plasmon-enhanced region of TERS is a small volume with a diameter of ca. 10 nm under the tip (Supplementary Fig. 16)^9,26,27,46^. We consequently estimate that only around 50 monomers were probed within the hotspot.

All time-resolved TERS measurements were carried out by positioning the Ag tip on a monomer ML, and monitoring the Raman signals over time at that location (Fig. 1d, e). Both plasmon-induced activation and Raman enhancement were simultaneously achieved by irradiating the tip with the same laser light. The excitation and collection of the Raman spectra were performed along the same optical pathway. The time-dependent variation of the TER spectra revealed significant chemical differences between the monomer and 2DP MLs (Fig. 2a, b and Supplementary Fig. 20). Under continuous laser illumination (633 nm) over 120 s, TERS results clearly indicate the occurrence of a plasmon-driven 2D polymerization (Fig. 2a, b), because the disappearance of the 1425/1445 cm^−1^ bands (breathing mode of the anthracene blades) is accompanied by the appearance and growth of the 965/975 cm^−1^ bands (C–C stretching of the newly formed bridgehead)^46,47^, and the resulting TERS features are almost the same as that in the 2DPs driven by UV light irradiation (365 nm, Fig. 2c, d).

However, the same LSP in the hotspot shows a lower catalytic activity for monomer **2** on Au(111), as its distinctive 975 cm^−1^ band (60 s) appears later than the 965 cm^−1^ band (20 s) for monomer **1** (Fig. 2a, b). That is probably due to a number of reasons: (i) the electron-withdrawing tetrafluorinated groups at the end of the blades of monomer **2** cause the frontier orbital energy to increase (Supplementary Figs. 21 and 22), thereby increasing the reaction barrier for the [4+4]-cycloaddition;^56^ (ii) the three hydroxymethyl legs in the middle ring of monomer **1** enable better spatial matching between two adjacent anthracene blades, thereby decreasing the spatial freedom of their face-to-face packing to facilitate polymerization into a 2DP; (iii) the three hydroxymethyl legs in the middle ring of monomer **1** promote the hot electron transfer from Au(111) to their reactive sites (in the middle ring), thereby increasing the probability of a plasmon-induce reaction to occur. Plasmon-driven photodamage (see Supplementary Note 3 for more details) for the MLs can also be observed from the appearance of D and G bands of amorphous carbon when the samples are irradiated for a longer time (e.g., up to 300 s, Supplementary Fig. 20), with higher incident power (e.g., 0.6 mW, Supplementary Fig. 23), or a shorter wavelength (e.g., 532 nm, Supplementary Fig. 24)^33^.

### Excitation mechanism of the plasmon-induced 2D polymerization

**Fig. 3:**
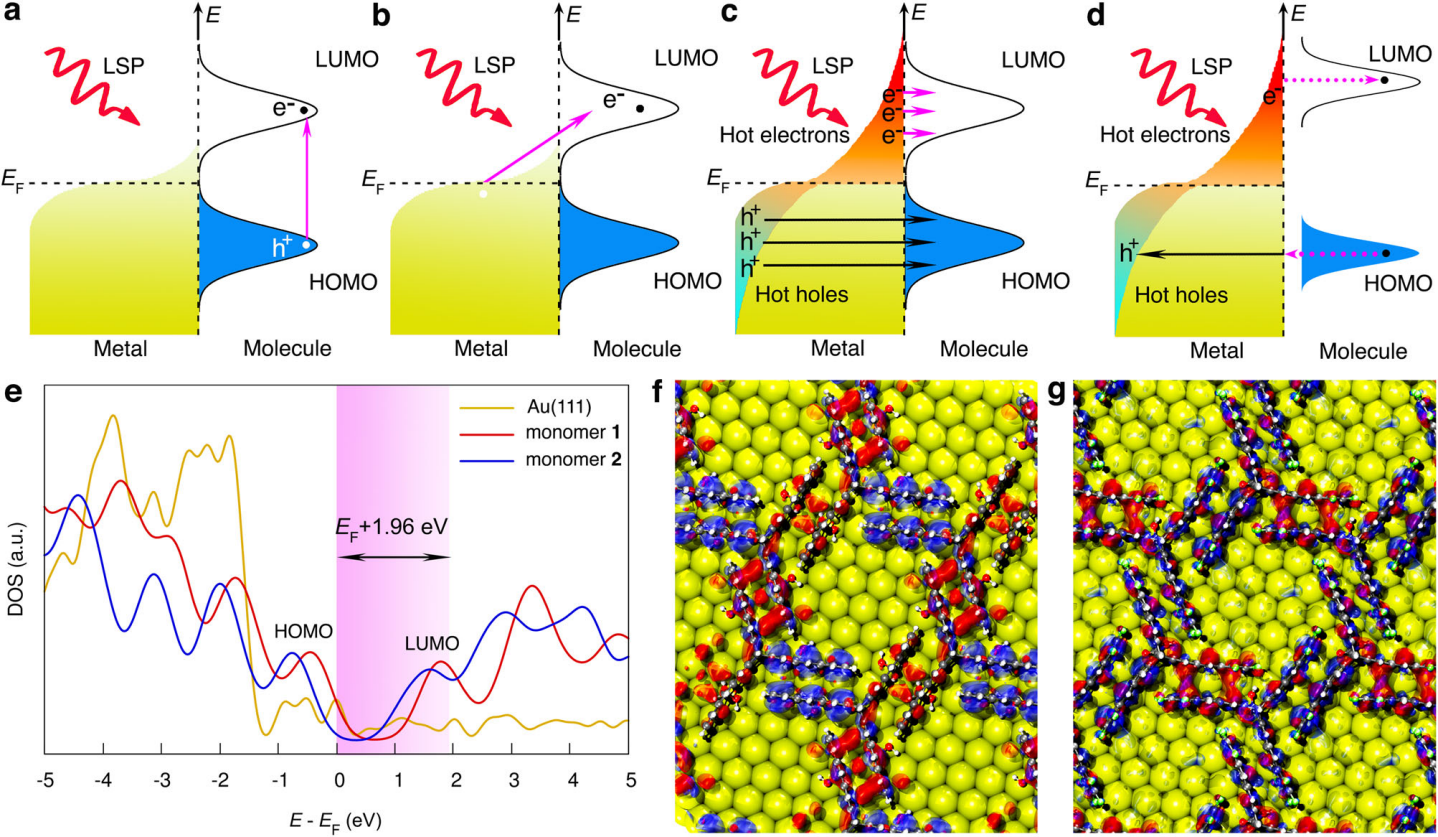
Excitation mechanisms for plasmon-induced chemical reactions. **a** Direct intramolecular excitation for a HOMO-LUMO transition. **b** Charge transfer mechanism from metal surfaces to adsorbate unoccupied states. **c**, **d** Indirect hot-carrier transfer from the metal surface to unoccupied states of the adsorbate **c** with and **d** without adsorbate/surface hybridization through inelastic tunneling processes. The localized surface plasmon (LSP) is a result of the strong coupling between metal-free electrons and incident electromagnetic field in the confined plasmonic system. Black and white dots indicate electrons (e^−^) and holes (h^+^), respectively. **e** The calculated projected DOSs of monomer **1** and **2** MLs on Au(111). The energy distribution of the hot electrons in the hotspots are highlighted. **f**, **g** The spatial distribution of the (blue) HOMO and (red) LUMO levels for monomer **f** **1** and **g** **2** MLs on Au(111), respectively. a.u. arbitrary units.

Several typical excitation mechanisms for PICRs with strong molecule/metal hybridization have been previously proposed, e.g., intramolecular excitation, charge transfer, vibrational excitation by local heat, and hot carrier transfer (Fig. 3a–d and Supplementary Fig. 25)^2,9^. These mechanisms were taken into consideration for our physisorbed system. The intramolecular excitation mechanism was excluded (Fig. 3a), because according to the calculated projected density of states (DOSs) of the MLs on Au(111), the energy gap between the highest-occupied molecular orbital (HOMO) and the lowest-unoccupied molecular orbital (LUMO) is ~2.31 and 2.42 eV for monomer **1** and **2** MLs, respectively (Fig. 3e). Hence, neither the incident laser energy nor the induced LSP (1.96 eV) is sufficient to excite a direct HOMO-LUMO transition for these monomers. The charge transfer mechanism was also ruled out, since it would require the resonance transient negative ion (TNI) states to be dissociative, which is not the case for the anthracene-based monomers (Fig. 3b)^9^. In addition, the LSP (1.96 eV) was too low to stimulate the direct charge transfer via the interband transition (from the top of the d-band to empty conduction p-band) near the *E*_F_ at ca. 2.4 eV and ca. 4.0 eV for Au and Ag, respectively^57^. The local heating is unlikely to play an important role to promote 2D polymerization (Supplementary Fig. 25), because the [4+4]-dimers formed would be thermally reversed to monomers^58^. Last, the tunneling electrons (*E*_F_ + eV_bias_ (0.1 eV)) even cannot vibrationally excite any reaction such as rotation, desorption, or dissociation of some molecules (Supplementary Fig. 25), let alone [4+4]-cycloaddition polymerization^9,59^.

For chemisorbed systems (Fig. 3c), hot carriers may drive all possible PICRs initiated from the formation of TNI states owing to their broad energy distribution (*E*_F_ ± *E*_LSP_)^60^. They are efficiently generated through non-radiative decay of LSPs in a confined nanoscale system^61^. According to FDTD calculations (Supplementary Figs. 16–19), the strongest LSP coupling can be excited in the TERS nanogap under 633 nm laser illumination, where hot electron-hole pairs will be created by surface-collision assisted absorption (Landau damping)^62^. In other words, collective oscillations of electrons in the localized hotspots enable strong absorption of the incident photons, creating a steady-state probability of the electrons with energies between *E*_F_ and *E*_F_ + 1.96 eV, and leaving the holes with energies between *E*_F_ and *E*_F_ − 1.96 eV, respectively^63^. Hot carriers then quickly redistribute their excess energy by electron–electron scattering processes towards a Fermi–Dirac distribution^63^. A three-step model can be considered for the indirect hot-electron transfer, absorption of photons, followed by the creation of hot carriers, and finally, transfer of electrons from metals to unoccupied molecular orbitals^60^. When strong hybridization exists between the metal Fermi level and the molecular orbitals of a chemisorbed adsorbate, such an interaction will facilitate hot-electron transfer and TNI states formation.

Importantly, hot electron transfer through an inelastic tunneling process can also occur in physisorbed MLs (Fig. 3d), even without strong interaction between the surface and MLs^64^. When the LSP is aligned with the molecular LUMO energy level near the metal Fermi level (Fig. 3e), the oscillating hot electrons could be injected into the molecular LUMO and thus generate the TNI and singlet excited states (*E*_F_ → *S*_1_). This process requires less energy than that *S*_0_ → *S*_1_ transition via photon excitation (e.g., ca. 2.97 eV for anthracene)^65^. After intersystem crossing, one of the anthracene-based blades may undergo a non-radiative transition to the triplet excited state (*T*_1_) and change its spin multiplicity. This enables matching of the orbital symmetry of the adjacent anthracene pair, and allows for the [4+4]-cycloaddition to occur^52^. However, due to the narrower spatial distribution of the LUMO for monomer **2** (Fig. 3f, g), and the better electron conduction from the three hydroxymethyl groups in monomer **1** (Fig. 1a), the relative efficiency of the hot electron transfer is expected to be lower in monomer **2** ML^66^. Consequently, the same hot electrons are harder to activate the 2D polymerization within monomer **2** ML (Fig. 2), although it shows a lower LUMO level on Au(111) than that of monomer **1** ML (Fig. 3e). In parallel, the hot electrons fail to revert the polymers back to the monomers simultaneously (Supplementary Figs. 26 and 27), because the LUMO levels of 2DP **1** (3.36 eV) and **2** (2.38 eV) MLs on Au(111) were calculated to be much higher than the LSP energy (1.96 eV).

To determine the major contributor for the hot carrier generation, shell-isolated nanoparticle-enhanced Raman spectroscopy (SHINERS) was employed by covering a ML of monomer **1** with Ag@SiO_2_ nanoparticles that were composed of a ca. 100 nm Ag core and a ca. 2 nm thin SiO_2_ shell. Under the same laser excitation conditions (633 nm, 0.3 mW), the time-dependent spectral evolution reveals that the 2D polymerization can also occur in SHINER hotspots as indicated by the appearance of the 965 cm^−1^ band (Supplementary Fig. 28). Since each Ag nanoparticle was isolated by the SiO_2_ insulating shell, the hot carriers must have been mainly excited on the Au(111) surface, in line with the previous studies^63^. Nevertheless, compared to the TERS system, such an Ag@SiO_2_/Au substrate configuration shows a lower efficiency for hot-carrier creation and thus a lower reactivity for 2D polymerization (Supplementary Fig. 28). This is probably due to the weaker plasmon resonance between the incident laser and the Ag@SiO_2_/Au configuration, caused by the larger size of the Ag nanoparticle and the bigger gap of the nanoparticle/substrate nanocavity than that in the TERS system^67^.

### Growth mechanism of the plasmon-induced 2D polymerization

**Fig. 4:**
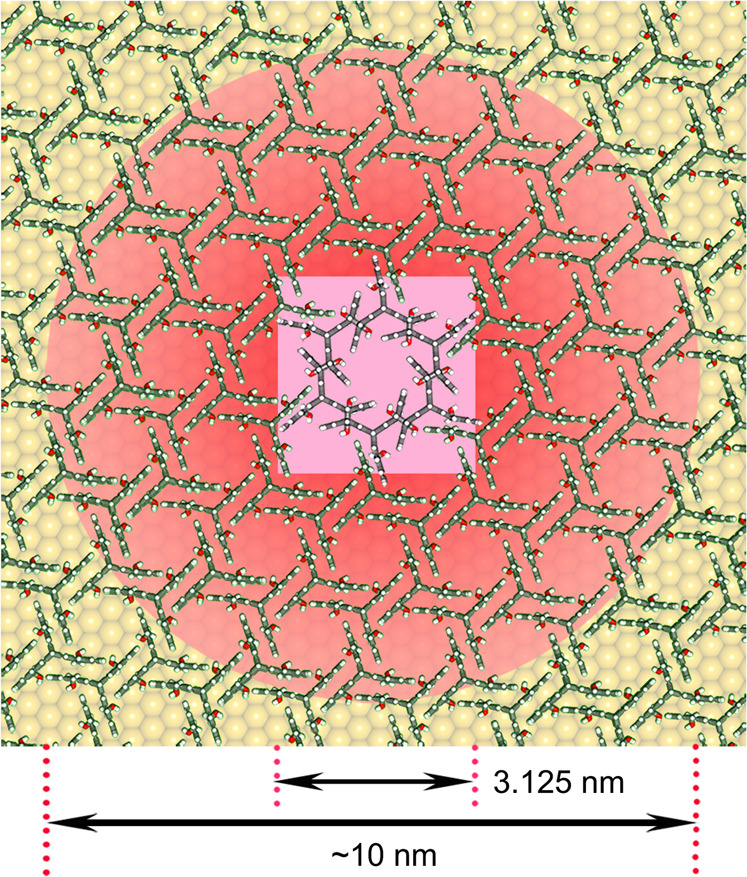
Schematic illustration of a monomer 1 packing model on Au(111). This model contains a single imaging pixel (pink square), a growth nucleus (reacted unit, highlighted in pink in the center), and the strongest hotspot area (red circle).

**Fig. 5:**
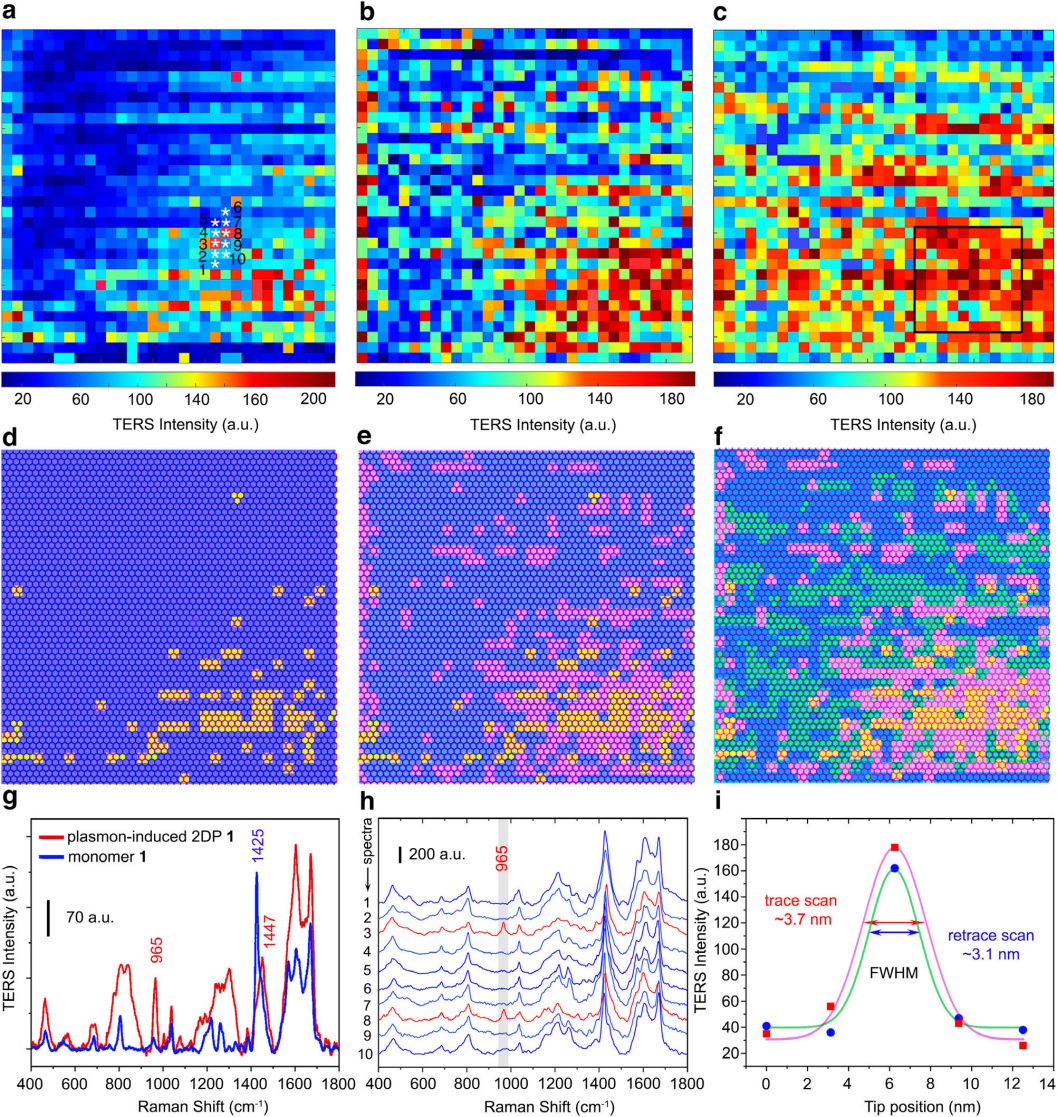
Consecutive TERS imaging of the plasmon-induced 2D polymerization within monomer 1 ML on Au(111). **a** The 1st, **b** 2nd, and **c** 3rd TERS intensity images of the band at 965 cm^−1^. The maps were repeatedly measured over a 100 × 100 nm^2^ area with 3.125 × 3.125 nm^2^ pixels resolution at the same location under continuous laser irradiation (633 nm, 0.3 mW, 10 s integration). **d**–**f** Corresponding growth kinetics of the plasmon-induced [4+4]-cycloaddition polymerization in monomer **1** on Au(111). In order to connect the TERS maps with the chemistry of the monomer array, an artificial hexagonal network was created considering relevant sizes of the monomer packing obtained from the model in Fig. 4. This network contains 55 × 55 hexagonal units. Lattice sites specified with blue areas represent the unreacted monomers and the yellow (1st imaging), pink (2nd imaging), and green (3rd imaging) areas exhibit polymerized parts based on the signal/noise criterion S/N > 4 of the band at 965 cm^−1^. **g** Baseline corrected average TER spectra of monomer **1** (blue curve, averaged from 1024 spectra in **a**) and corresponding plasmon-induced 2DP **1** (red curve, averaged from 100 spectra in the black frame in **c**). **h** 10 sequential TERS measurements along the line trace and retrace scans marked in **a**. **i** Corresponding TERS intensity and Gaussian fitting of the band at 965 cm^−1^ in **h**. The spatial resolution is estimated using a full-width at half maximum (FWHM) analysis. a.u. arbitrary units.

Recently evidence was gained concerning the mechanisms of polymerizations in layered single crystals to give 2D polymers^44^. It was therefore of obvious interest to see whether the same question could be studied by TERS for monomers adsorbed on a metallic substrate. For this purpose, we repeatedly collected TERS images of monomer **1** on Au(111) from 100 × 100 nm^2^ maps with 32 × 32 pixels at the same location as far as the thermal drift allowed. This drift is mostly random but some directionality cannot be excluded and it is estimated to be on the order of a nanometer per minute. According to a DFT-optimized monomer packing model (Fig. 4), each pixel (3.125 × 3.125 nm^2^) should cover 6 monomer molecules **1** completely that are hexagonally arranged around a pore and, including neighboring monomers, contain 6–12 anthracene-anthracene pairs (pink square), six of which are shown in dimerized form (growth nucleus). First, the experimental parameters, i.e., incident wavelength, laser power, and integration time, were optimized for better long-term TERS imaging and minimally invasive characterization(Supplementary Figs. 23 and 24). As depicted in Fig. 5a–c and Supplementary Fig. 29, the characteristic 965 and 1425 cm^–1^ bands for the polymer and the monomer, respectively, were chosen to produce the Raman intensity images. These complementary maps further confirmed the appearance of new [4+4]-dimers and the absence of unreacted anthracene blades after the plasmon-mediated catalysis (Fig. 5a–c and Supplementary Fig. 29).

Because the images in Fig. 5a–c do not reflect the molecular scale, in the next step we created an artificial hexagonal network based on the model from above. This allowed us to compare the TERS findings with chemical reality. Those parts where reactivity was observed were given a particular color to indicate local polymerization. Figure 5d shows such a map where blue indicates the area of no reaction and the yellow dots reflect where some dimerization has occurred. Note that this representation is not quantitative in the sense that all monomer molecules contained in a yellow area must have actually reacted with one another. Recording of this map took about 3 h. After that time a second map was continuously recorded of the same sample. Although thermal drift may pose a problem, we succeeded in overlaying the first and second map to see whether there is any correlation between the reacted parts in both. This overlay is shown in Fig. 5e, where the yellow color refers to areas where the reaction had occurred during the first scan and the pink color refers to the same of the second scan. The overlay was done such that the contact between yellow and pink areas was maximized. Interestingly, most of these areas could actually be brought into contact, which suggests that we encounter a phenomenon of chemical reality. It appears as if the pink areas are preferentially formed in direct proximity to the already existing yellow ones. Given this finding, we moved along the same lines by creating a third map which resulted in a total scanning time of about 9 h. Again, an overlay of the third map with the map in Fig. 5e was created, resulting in Fig. 5f. Now the newly reacted areas are presented in green color. As can be seen, the third map can be adjusted to Fig. 5f in a way that most of the green parts are in tight contact with either yellow or pink areas. This again suggests that thermal drift is not pronounced enough to obscure what seems to happen chemically, namely that an existing polymerized part triggers new growth at its edges. Although there is no proof of direct chemical connection between yellow, pink, and green areas, it appears that our experiments point towards a self-stimulating growth mechanism. It may be of interest to mention that in single crystals most 2D polymerizations follow the self-inhibited growth mechanism and that we therefore may see an effect of the metallic substrate on polymerization here (see Supplementary Note 4 for more details).

Whenever performing a polymerization, the conversion number plays an important role. In UV-driven 2D polymerization such numbers have been determined using TERS. For example, the polymerization of monomer **1** performed at the air/water interface after 1 h UV-irradiation gave a conversion number of 87.7 ± 1.8% (Supplementary Fig. 30)^46^. In a similar study on Au(111) upon 6.5 h UV light excitation, a conversion of 92 ± 12% was reported^45^. Although it was not in the focus of the current work to determine the efficiency of plasmon-induced [4+4]-cycloaddition polymerization, some preliminary insights into this question could nevertheless be gained. For that purpose, a 31.25 × 31.25 nm^2^ area in Fig. 5c was selected (black frame, 10 × 10 pixels), in which the conversion appeared to be the highest. Compared the averaged TER spectrum of monomer **1** with that of plasmon-induced 2DP **1** (Fig. 5g), data evaluation afforded a conversion number of 80.1 ± 2.3% (Supplementary Fig. 30 and Supplementary Table 1). We expect that further TERS scanning would lead to even higher values.

Notably, this in-plane polymerization on Au(111) can be realized and recorded in real space within individual map pixels under ambient conditions (3.125 × 3.125 nm^2^, Fig. 5h, i). The area per pixel is smaller than the plasmon-enhanced hotspots (ca. 10 nm in diameter, Supplementary Fig. 16) and far beyond the spot size of the laser focus (ca. 2 μm in diameter). This implies that only certain hot carriers match the molecular LUMO level (over *E*_F_ + 1.86 eV, Fig. 3e) and thus overcome the reaction barrier. In contrast to the calculated mean free path (ca. 50 nm) of hot electrons on Au/Ag surfaces and the measured transport distance (ca. 20 nm) of similar hot carriers^61,63^, the spatial reach (ca. 3.125 × 3.125 nm^2^) of the hot electrons is much smaller in the plasmon-induced [4+4]-cycloaddition system (Fig. 5d), since hotter carriers can only be collected within smaller regions in hotspots before their redistribution and decay^63^. In other words, hot electron lithography may be used to “print” a single 2DP **1** unit (ca. 2.234 nm in size) within a well-arranged monomer ML during TERS imaging with a step size of 3.125 nm (Fig. 4). Alternatively, without assistance from the resonant plasmon, only intense UV light irradiation (365 nm), rather than the red (633 nm) or green (532 nm) laser lines, was able to efficiently trigger [4+4]-cycloaddition on Au(111) (Supplementary Fig. 31)^45^.

In order to examine the spatial resolution, Fig. 5h plots the TER spectra acquired in Fig. 5a during line trace and retrace, across individual 2DP **1** growth areas in the monomer arrays. The TERS intensity variation of the representative 965 cm^−1^ band with the tip position is shown in Fig. 5i. Unlike STM imaging (Supplementary Fig. 32 and Supplementary Note 5), TERS imaging provides rich chemical information and distinct reaction contrast for the molecular MLs at the nanoscale (Fig. 5d–f). The line profile analysis suggests a lateral resolution of ~3.7 nm, estimated by a full-width at half maximum (FWHM) analysis of the Gaussian fit (Fig. 5i). Occasionally, a plasmon-induced 2DP nanoribbon could be obtained from certain locations in monomer **1** ML when sufficient hot carriers were generated (0.6 mW, 10 s integration, Supplementary Fig. 33). This may shed a light on the nanolithography of 2DP monolayers by manipulating the plasmon-induced [4+4]-cycloaddition reaction (see Supplementary Note 6 for more details)^36,49,68^.

## Discussion

In conclusion, we visualized photon–electron molecule interactions during plasmon-induced [4+4]-cycloaddition polymerization of anthracene-based monomers on Au(111) via TERS imaging in real-time and space. Amphiphilic monomers **1** and **2** with the same anthracene-based backbone but different substituents were utilized for forming the corresponding 2DPs exciting both by UV laser irradiation (365 nm, 3.40 eV) and hot carriers (*E*_F_ + 1.96 eV). The monomer MLs were prepared at an air/water interface on a LB trough, which allowed for the controllable transfer of those MLs onto Au(111) with physisorbed molecule/substrate interactions. The same TER spectral features of the plasmon-induced 2DPs as those produced by UV light irradiation were found. However, the different substituent and spatial effects from their molecular structures were shown to affect the catalytic activity of the hot carriers. Our TERS imaging observed the in-plane polymerization within individual map pixels (3.125 × 3.125 nm^2^) and resolved single 2DP **1** growth areas with ~3.7 nm spatial resolution. Combined with DFT calculations, we propose that the 2D polymerization is initiated from the formation of TNI and triplet excited states, and that hot electron transfer occurs from Au(111) to the MLs through an inelastic tunneling process when the LSP energy matches the molecular LUMO levels. Applying hot carriers to these MLs in consecutive scans supports the view that initially formed areas with polymer triggers further polymer growth in their direct proximity, which would be the characteristics of a self-stimulating growth mechanism. This work extends the mechanistic understanding of PICRs and shows a way for localized design at the nanometer scale and molecular level of covalent 2D materials.

## Methods

### LB monolayer preparation and transfer

Amphiphilic monomers **1** and **2** were synthesized according to our previous work^46,47^. Langmuir experiments were carried out on a Langmuir–Blodgett trough (KSV 2000 System 2, Finland) equipped with a platinum Wilhelmy balance and a dipper module. The trough is made from Teflon and the barriers from Delrin. The surface pressure was measured through the Wilhelmy balance with a precision of 0.01 mN/m. Prior to each experiment, the trough was cleaned with a sequence of millipore H_2_O, CHCl_3_, EtOH, millipore H_2_O, meanwhile wiped dry using dust-free papers (Kimtech Science Precision Wipes, Kimberly-Clark Professional, Woodbridge, Ontario, Canada) after each solvent treatment. Afterward, it was given a final rinse with millipore H_2_O. The barriers were cleaned with millipore H_2_O, EtOH, and then again millipore H_2_O, meanwhile wiped off with the dust-free papers after each solvent treatment. Afterward, they were given a final rinse with millipore H_2_O. The dipper stage carrying the substrate (silicon wafer or Au(111)) was immersed into the water before the compress experiment (transfer from below). 60–80 μL of monomer stock solutions (0.5 mg/mL in CHCl_3_) were applied for the monolayer preparation using a 100 μL glass microsyringe. After delaying 30 min to evaporate the solvent on the trough, the barriers were compressed at a speed of 3 mm/min to a surface pressure of 20 mN/m. UV-driven photopolymerization on the trough was carried out using LEDs of wavelength *λ* = 365 nm (Omicron Laser LEDMOD365.250.OEM) after the monolayers had been kept at a surface pressure of 20 mN/m for 1 h. Both monomer and 2D polymer monolayers were transferred by the Schäfer technique^46,47^. The presubmerged substrate (silicon wafer or Au(111) substrate) was pulled up by the dipper at a constant rate of 0.5 mm/min. After drying at room temperature, the collected monomer/2D polymer monolayers were subjected to optical microscopy, SEM, AFM, and TERS measurements.

### Raman and TERS measurements

All Raman/TERS and STM measurements were performed under ambient conditions on an integrated STM/Raman microscope system (Ntegra Spectra, NT-MDT, Zelenograd, Russia), which is placed in a homemade acoustic isolation box. A standard neon lamp (Renishaw, UK) was used to calibrate the spectrometer. TERS probes were made from electrochemically etched silver wires (diameter 0.25 mm, 99.9985% purity, Alfa Aesar) according to our previous work^38,39^. TERS maps were collected in the STM feedback mode (constant current), the sample surface was modulated by the piezo stage in the *x*, *y*, and *z* directions with the relative laser-to-tip position fixed. A 632.8 nm laser wavelength with a local power density of 0.3 mW was chosen as the excitation source. All spectra were recorded after an exposure time of 5 s and were averaged by 2 repetitive accumulations. TERS imaging was set under a tunneling condition of 100 pA and 100 mV to avoid scratching the monolayers. All TERS images were collected after overnight measurements (continuous laser illumination and data recording) to maximally decrease the drift of the system to ∼1 nm/min. For high-resolution STM imaging, the bias voltage of 100 mV and tunneling current of 1.0 nA were applied after TERS imaging. Finally, all spectral data were processed according to previously described procedures (Supplementary Figs. 34 and 35)^38,39^.

### FDTD and DFT calculations

The spatial distribution and absorption spectra of the electric field at the Ag tip/Au substrate nanogap were calculated using the FDTD method (Lumerical Solution). The simulation model comprised an electrochemically etched Ag tip (curvature radius *r* = 50 nm, cone angle *θ* = 40°, Supplementary Fig. 15), an Au metal slab, and a vacuum layer. The tip-surface distance was set at 1 nm, and the incident wavelength was set at 633 nm. The dielectric function of Au and Ag was taken from the experimental data reported by Johnson et al.^69^. A plane-wave has been employed on the nanogap with different incident angles (0°, 45°, and 90° to the substrate surface normal, Supplementary Figs. 16–19). This wave was polarized along the *x* direction and propagated along the *z* direction. In order to obtain the absorption spectra of the LSP, we fixed the incident angles while tuning the incident wavelengths of polarized light applied to the Ag tip/Au substrate nanogap (Supplementary Figs. 16–19)^55,70^.

The projected density of states (DOSs) and the HOMO/LUMO calculations were performed using the CP2K package^71^. PBE functional with Grimme D3 correction was used to describe the system^72,73^. Kohn–Sham DFT has been used as the electronic structure method in the framework of the Gaussian and plane waves method^74,75^. The Goedecker–Teter–Hutter (GTH) pseudopotentials were applied in the system^76,77^. The TZV2P-MOLOPT-SR-GTH basis sets were utilized to describe molecules and the DZVP-MOLOPT-SR-GTH basis sets were utilized to describe Au(111) surfaces^74^. A plane-wave energy CUTOFF of 400 Ry has been employed. The Fermi–Dirac smearing has been applied to study the metallic system, and the electronic temperature is set to 300 K. The gold surface containing 172 metal atoms was shaped to obtain (111) surfaces with four ($\sqrt{43}$ × $\sqrt{43}$) atomic layers within a hexagonal 19.34 × 19.34 × 30 Å^3^ box. All the simulations are carried out by keeping the two bottom metal layers fixed at the initial coordinates in order to maintain the bulk behavior of the inner part of the slab.

## Supplementary information

Supplementary Information

## Data Availability

The original data used in this publication are made available in a curated data archive at ETH Zurich (https://www.research-collection.ethz.ch) under the 10.3929/ethz-b-000495638.
