# Supplementary material for: In-situ nanospectroscopic imaging of plasmon-induced two-dimensional [4+4]-cycloaddition polymerization on Au(111)
Source: Nat Commun. 2021 Jul 27;12:4557. doi: 10.1038/s41467-021-24856-5 (PMC8316434; doi:10.1038/s41467-021-24856-5)
Supplement: Supplementary file 1 — Supplementary Information [file 41467_2021_24856_MOESM1_ESM.pdf]

SUPPLEMENTARY INFORMATION

In-situ Nanospectroscopic Imaging of Plasmon-induced Two-dimensional [4+4]- Cycloaddition Polymerization on Au(111)

Feng Shao,[1, 2*] Wei Wang,[3] Weimin Yang,[4] Zhilin Yang,[4] Yao Zhang,[5] Jinggang Lan,[6*]
A. Dieter Schlüter,[7] Renato Zenobi [2*]

[1] *Department of Physics and Astronomy, National Graphene Institute, University of Manchester, Manchester, M13 9PL, UK*

[2] *Department of Chemistry and Applied Biosciences, ETH Zurich, Vladimir-Prelog-Weg 3, 8093 Zurich, Switzerland*

[3] *Shanghai Key Laboratory of Green Chemistry and Chemical Processes, School of Chemistry and Molecular Engineering, Chang-Kung Chuang Institute, East China Normal University, 3663 N. Zhongshan Road, Shanghai 200062, P. R. China*

[4] *Department of Physics, Collaborative Innovation Center for Optoelectronic Semiconductors and Efficient Devices, Jiujiang Research Institute, Xiamen University, Xiamen, Fujian 361005, P. R. China*

[5] *Hefei National Laboratory for Physical Sciences at the Microscale and Synergetic Innovation Center of Quantum Information & Quantum Physics, University of Science and Technology of China, Hefei 230026, China*

[6] *Department of Chemistry, University of Zurich, 8057 Zurich, Switzerland*

[7] *Department of Materials, Polymer Chemistry, ETH Zurich, Vladimir-Prelog-Weg 5, 8093 Zurich, Switzerland*

**Corresponding Authors: feng.shao@manchester.ac.uk; jinggang.lan@chem.uzh.ch; zenobi@org.chem.ethz.ch*

Table of Contents

1. Supplementary Notes

2. Supplementary Figures

3. Supplementary Methods

3.1 Langmuir-Blodgett (LB) Trough and Monolayer (ML) Preparation
3.2 Langmuir-Blodgett (LB) Film Transfer and Spanning
3.3 Scanning Electron Microscopy (SEM)
3.4 Atomic Force Microscopy (AFM)
3.5 UV-Vis Spectroscopy
3.6 Raman and TERS measurements
3.7 FDTD Calculations
3.8 DFT and TERS Calculations
3.9 Conversion Ratio Calculations
3.10 Spectral Processing

4. Supplementary References

1. Supplementary Notes

Supplementary Note 1

UV-driven [4+4]-cycloaddition polymerization in the monomer MLs

In our previous work, we demonstrated that the UV-driven [4+4]-cycloaddition conversion is feasible for creating 2DPs at the air/water interface.[1, 2] In contrast to the monomer MLs, the 2DP sheets obtained can span TEM grids with 20 × 20 μm^2 holes, as illustrated by scanning electron microscopy (SEM) images (Supplementary Fig. 7). This confirms the mechanical stability and homogeneity of the irradiated 2DPs. The thickness of all MLs was measured with tapping mode atomic force microscope (AFM), and consistently found to be ca. 0.8 nm over micron-sized areas (Supplementary Figs. 8-11). Compared to the monomer models (ca. 0.6 nm, Supplementary Fig. 1), the additional thickness of ca. 0.2 nm in the LB MLs suggests a water layer hydrogen-bonded to the hydrophilic tail groups.

To gain insight into the UV-driven [4+4]-photocycloaddition, confocal Raman/TER spectra were recorded from the monomer powders and monomer/polymer MLs. The Raman peak assignments were supported by DFT calculations (Supplementary Fig. 13). The typical anthracene breathing mode at 1425 /1445 cm^{-1} occurs both in the powders and MLs of monomers **1** and **2**, respectively (Supplementary Fig. 13). These bands disappear virtually completely or shift to 1447 cm^{-1} upon UV irradiation, while new characteristic 965/975 cm^{-1} bands appear in the 2DPs **1** and **2** samples, respectively. They are assigned to the bridgehead C-C stretching vibrations of anthracene [4+4]-dimers.[1] The simultaneous disappearance and appearance of these particular bands is an unambiguous indication for the occurrence of polymerization and prove the crosslinking based on anthracene [4+4]-photocycloaddition.[1, 2]

Supplementary Note 2

Electromagnetic field enhancement and hot carrier generation

It is well known that LSPs in hotspots can induce highly enhanced electromagnetic fields, charge transfer, local heat generation, as well as hot carrier excitation.[3, 4, 5, 6] For near-field Raman spectroscopy (e.g., TERS), a stronger local electromagnetic field will benefit a higher Raman signal enhancement, thereby enabling in-situ monitoring of reaction processes with high sensitivity. In our gap-mode TERS, if the tip material and geometry are adapted to the substrate material and the incident wavelength, strong antenna resonances can be formed and the highest field enhancement will be expected (Supplementary Figs. 16-19).[7] For plasmon-induced chemical reactions (PICRs), the generation of more hot carriers with higher energy will contribute to more efficient chemical transformations.[8] In this case, the hot carriers' energy is mainly related to the incident wavelength, while their population is mostly related to the efficiency of the plasmon resonance. Accordingly, one promising strategy to study PICRs in the tip-sample gap is to find out the right balance between electromagnetic field enhancement and hot carrier generation.

By using LSPs in the TERS hotspot both as the reaction trigger and signal readout for studying PICRs at the same time, we are able to: (i) achieve the highest field enhancement and the most efficient hot-carrier generation in the specific gap-mode configuration; (ii) carry out both reaction monitoring and chemical transformation within once TERS imaging scan.[7] Alternatively, another attractive way is to separate these two processes selectively by applying different incident wavelengths for individual purposes. For example, we can carry out a first "writing" scan to accurately fabricate molecular patterns under one laser irradiation, followed by a second "reading" scan to non-destructively check these previously written patterns under another laser irradiation. However, there are the following challenges with this approach: (i)

System compatibility. We have to choose a proper molecular model (e.g., specific LUMO level, Fig. 5) to match the first incident wavelength (e.g., the shorter one), while choosing a fitting gap-mode configuration (the tip/substrate material and geometry) to match the second incident wavelength (e.g., the longer one). (ii) Thermal drift. For TERS under ambient conditions, the thermal drift of the setups is unavoidable, estimated to be on the order of a nanometer per minute. Consequently, we might not scan the exact same location during these two processes (see Supplementary Note 5).

Supplementary Note 3

Plasmon-induced photodamage during the TERS imaging

LSPs in confined volumes (hotspots) lead to highly enhanced electromagnetic fields, charge transfer, local heat generation, as well as hot carrier excitation. [3, 4, 5, 6] These effects can open novel photoelectrocatalytic reaction pathways, which are not accessible thermally. For example, plasmon-induced photodamage is a general but undesired effect in SERS and TERS experiments. These chemical transformations in hotspots are perceived as sample degradation and dissociation due to the high energy of hot carriers,[9] of which energies are in excess of a certain threshold. These induced products (e.g., amorphous carbonaceous) and side reactions (e.g., desorption of molecular fragments) are similar to that known from X-ray or e-beam-mediated surface chemistry, despite more than 100-fold energy difference between visible and X-ray photons.[9]

Supplementary Note 4

Self-impeding and self-stimulating mechanisms

In a previous review,[10] Schlüter and co-workers analyzed Bragg and diffuse X-ray scattering of monomer/2Dpolymer single crystals for various conversion numbers, ranging from about 15-100%. Irrespective of what happens on the local scale, effective global strain distribution by self-impeding (or random) propagation mechanisms is ideal for 2D polymerization to happen smoothly. This means that the distance of unreacted anthracene pairs increases slightly in the direct vicinity of a site where dimerization has occurred, thus lowering the probability of a second reaction next to where the previous one took place. Other 2D polymerizations in single crystals were also analyzed but not to this level of scrutiny. Except for one case with peculiar packing likely renders polymerization propagation to be self-stimulating, in all other cases the self-impeding mechanism was likely folloed.[28] In the exceptional case, the layer had to undergo enormous rearrangements to make polymerization possible at all. These rearrangements, which involve all three dimensions, bring unreacted anthracene pairs located next to a reaction site in a concerted action closer together than normal, which in turn increases the probability of dimerization. This is the reason why this particular monomer follows the self-stimulating mechanism.

It is difficult to make a clear statement in our current 2D polymerization on Au(111) case, as the interplay between monomer adlayer and Au(111) substrate is not known. Given the above insights, although solely referring to single crystals, it is reasonable to assume to first approximation that the interactions between adlayer and substrate are responsible for bringing unreacted anthracene pairs near a reaction site somewhat closer together. However, whether the two lattices (monomer and Au(111)) are commensurate to one another, which would likely be a condition for the validity of such an assumption, is not known. Compared to the above mentioned exception, which solely refers to arguments forming a rationalization chain, the current work is the first experimental proof for self-stimulating growth in a polymerization leading to a 2D polymer. Such growth results in many polymer phases growing inside a

'dispersing' monomer phase and forming growth fronts encircling each polymer phase, separating it from the surrounding monomer. These growth fronts are expected to be seen from HR-AFM or HR-STM imaging. However, it is still a massive challenge to obtain STM/AFM imaging of the 2DP sheets with molecular resolution under ambient conditions (see Supplementary Note 5).

Supplementary Note 5

STM imaging of the 2DP monolayers under ambient conditions

Structural analysis of 2DP monolayers is exceedingly challenging because of the very small thickness and the tiny amount of material.[11] This has the consequence that most analytical methods are not sensitive enough and causes problems with sample preparation and handlings, such as the folding/distortion of the 2D sheets and the irregular undulation between the sheets and substrates. Several groups have intensely tried to reveal the periodic structures of 2DPs by AFM or STM imaging at the molecular level, yet only two successful cases on HOPG have been reported by Murray et al.[12] and Müller et al.[11]

For our TERS setup under ambient conditions, although it allows for obtaining chemical information of 2DPs at the nanoscale, the Ag tip used for TERS is not optimized to obtain atomic-resolution STM images. To avoid scratching the covalent sheet, the STM parameters are set to keep a relatively large distance between the tip and sample. Furthermore, thermal drift plays a role during our STM and TERS imaging. Consequently, it is difficult to obtain STM or TERS images of the 2DP monolayers at the molecular level. Regarding the way forward to achieve a higher resolution and to associate microscopic images with TERS results, AFM/STM and TERS imaging should be carried out in a well-controlled environment in the future, e.g., under ultra-high vacuum and at cryogenic temperature.

Supplementary Note 6

Plasmon-induced nanolithography of molecular monolayers

Nanolithography can create nanoscale patterns on different media, e.g., on silicon wafers and molecular monolayers, used in various fields of technology from electronic to biomedical devices.[13, 14] Our current plasmon-induced nanolithography is potential to write new 2D patterns onto a molecular monolayer. In order to obtain more custom-designed 2D organic patterns, we need to combine photochemistry and surface monolayers with TERS techniques subtly. For the plasmon-induced [4+4]-cycloaddition polymerization system, more practical parameter optimization, such as the molecular structure, the incident wavelength, the tip/substrate material and geometry, and the scanning sequence, should be taken into consideration before realizing custom-designed nanolithography.

The key for the successful fabrication of 2D organic patterns is usually bound with a suitable molecule design that can restrict its rotation in the monolayer and allow the occurrence of photopolymerization in the plane. The self-stimulating nature however only means that anthracene dimers next to a reaction site are moved a bit closer together. This makes them more amenable for reaction but does not result in reaction. Bond formation still needs to be triggered. Moreover, the spatial selectivity with which one can trigger dictates the spatial selectivity of the chemical response. Besides the proposed [4+4]-cycloaddition, several possible reaction candidates may be used in plasmon-induced nanolithography, e.g., [2+2]-cycloaddition.[15] Our new findings will be reported in due course.

2. Supplementary Figures

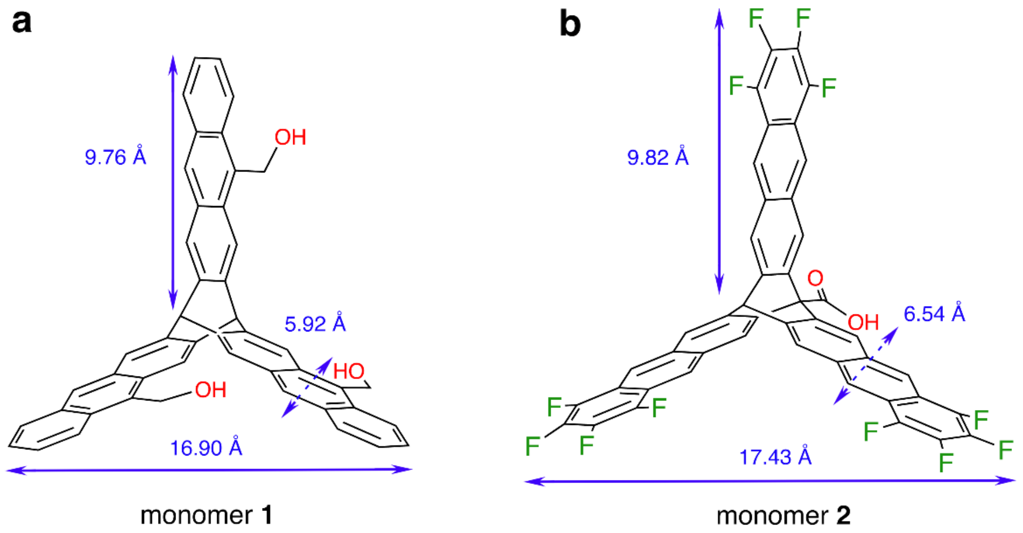

Supplementary Figure 1 | Chemical structure and dimensions of the designed monomers.

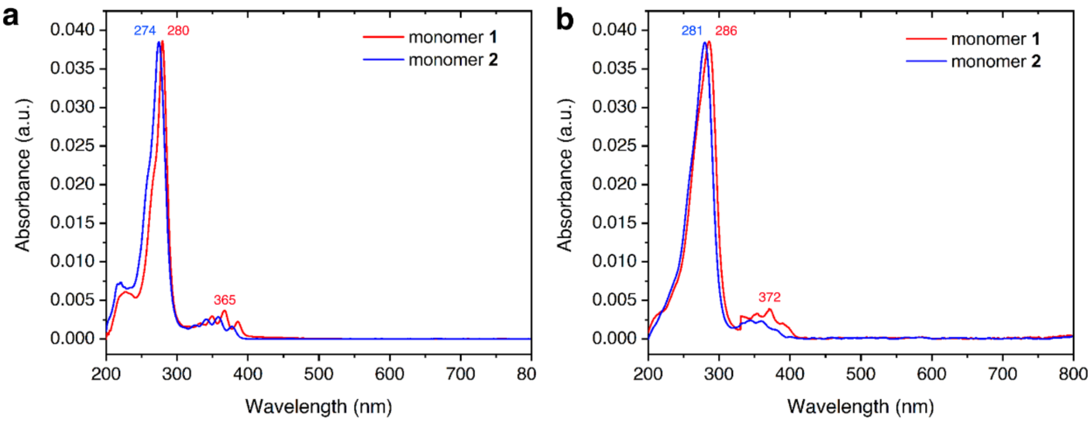

Supplementary Figure 2 | UV-Vis absorption spectra of the monomers (a) dissolved in acetonitrile and (b) deposited on quartz as LB monolayers by the Schäfer technique.[1] The red-shift of the adsorption in LB monolayers can be ascribed to their face-to-face stacking between neighboring anthracene pairs. a.u., arbitrary units.

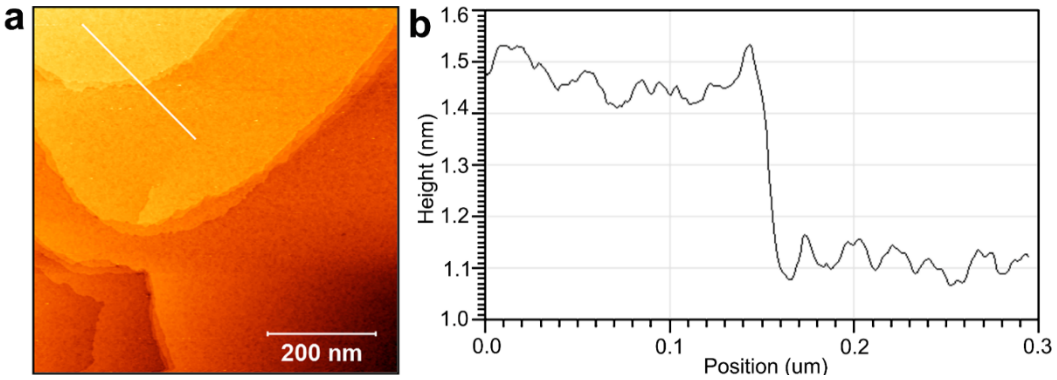

Supplementary Figure 3 | (a) STM image and (b) corresponding profile of the bare Au(111) surface with mechanically cut Pt/Ir tip. Single crystal terraces with atomically flat areas and single-atom steps were observed in the cross-sectional profile. The bias voltage was 0.2 V, and the tunneling current was 1.0 nA.

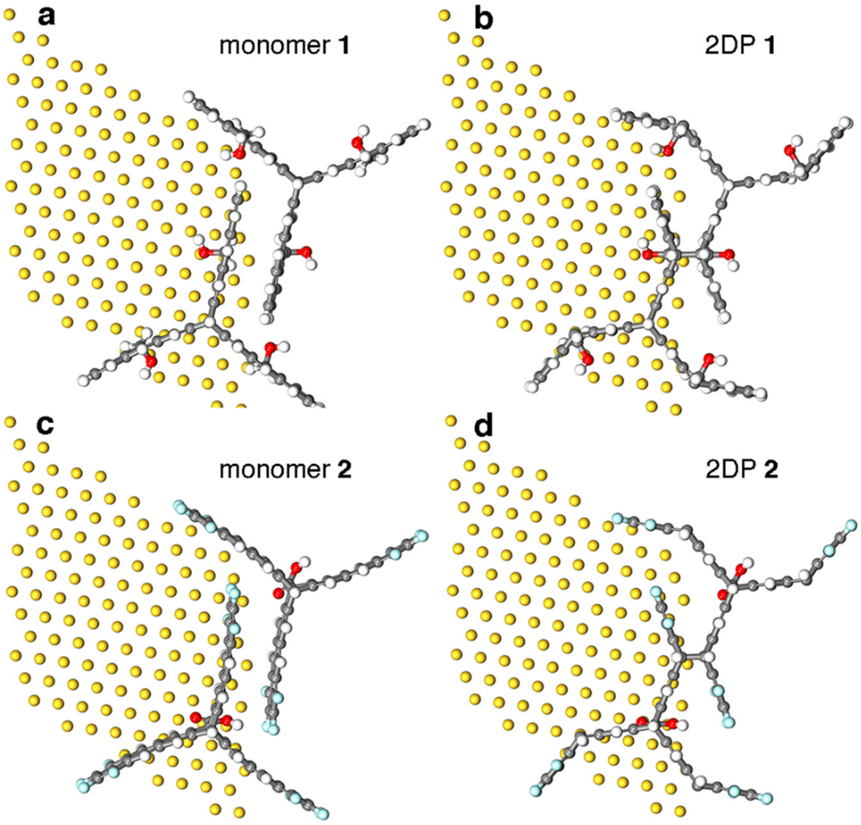

Supplementary Figure 4 | Top view of the DFT optimized structure units.

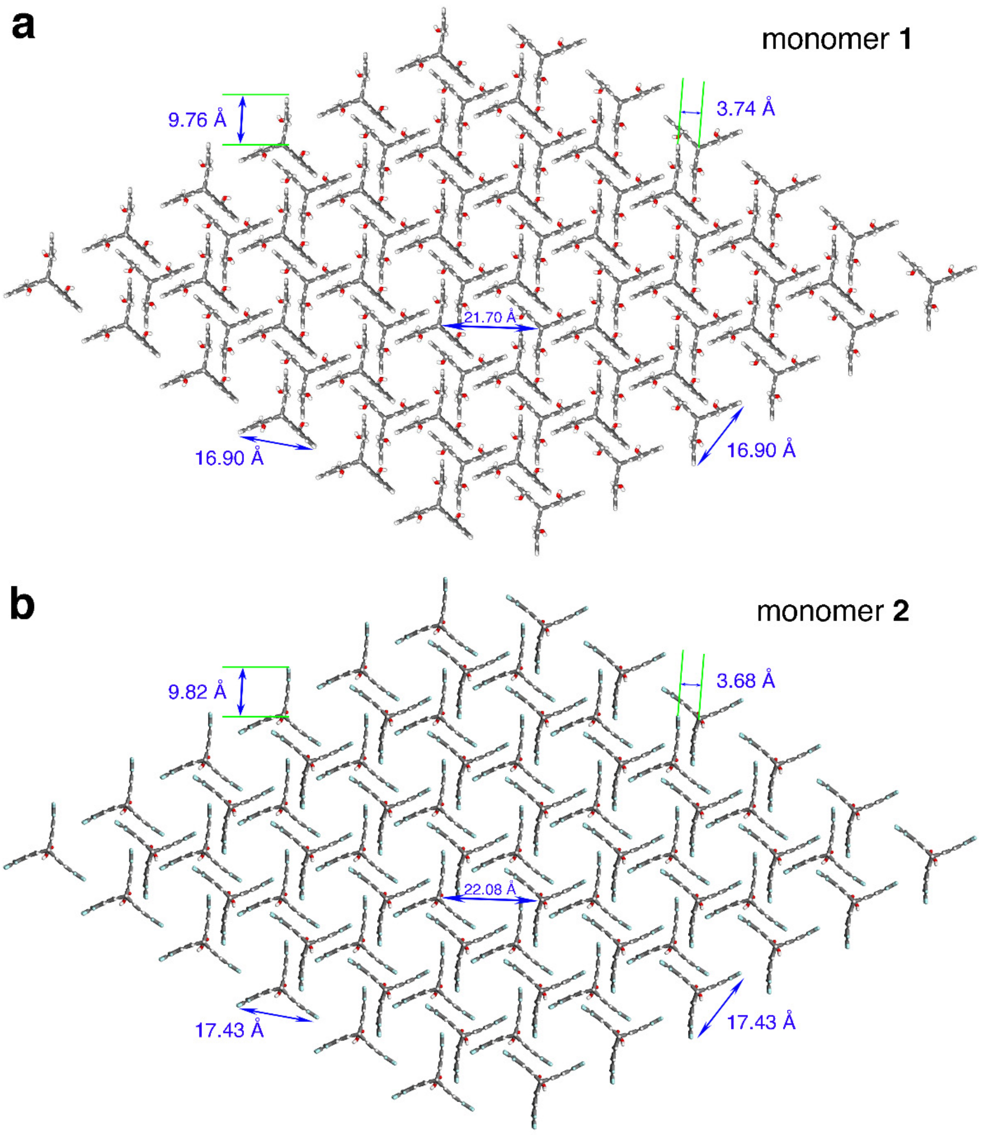

Supplementary Figure 5 | DFT simulated packing configuration and dimensions of (a) monomer **1** and (b) monomer **2** MLs on Au(111). These face-to-face packings have π-π stacking arrangements and give the calculated mean molecular area of (a) 153 $Å^2$ and (b) 158 $Å^2$ per molecule, respectively.

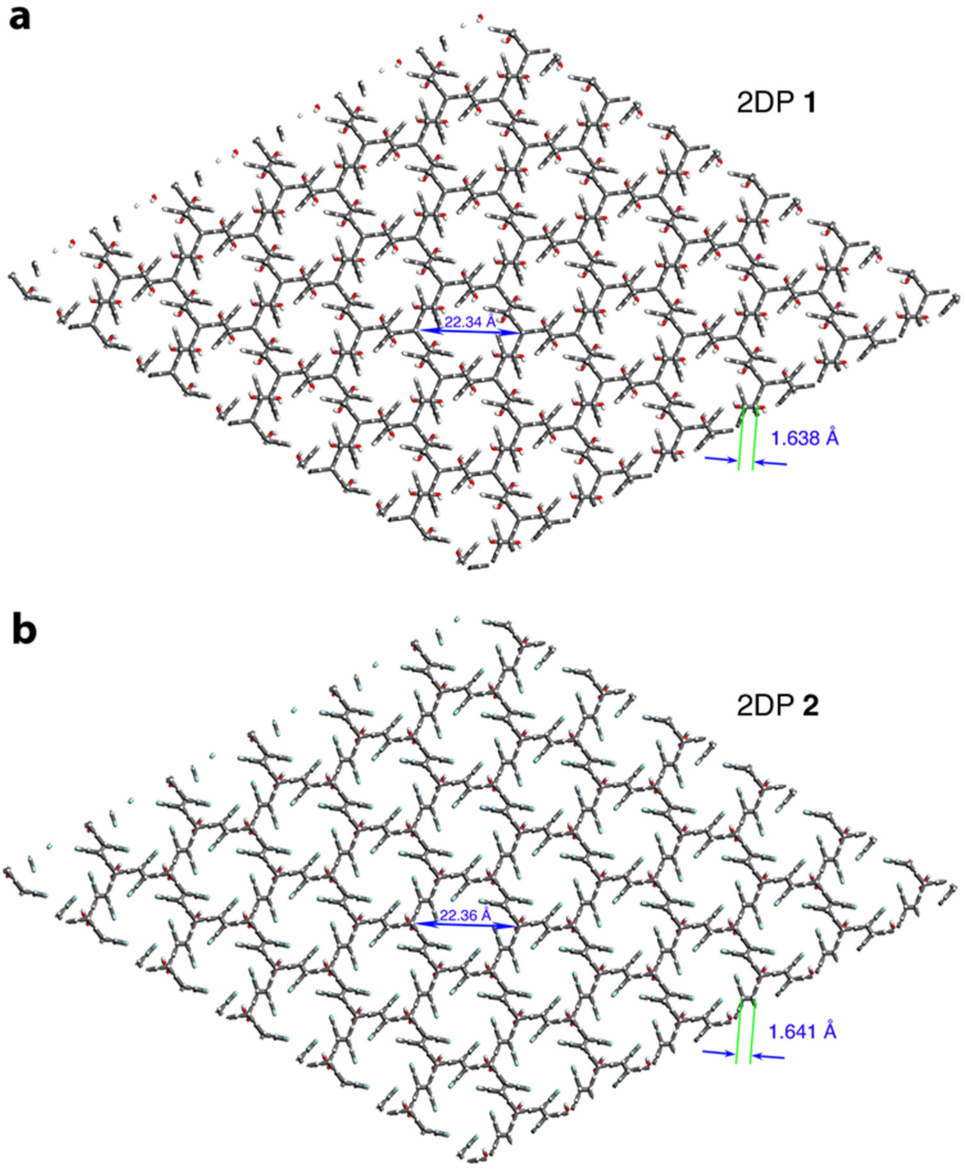

Supplementary Figure 6 | DFT simulated packing configuration and dimensions of (a) 2DP **1** and (b) 2DP **2** MLs on Au(111). These packings give a similar calculated mean molecular area of 162 Å^2 per molecule for the 2DPs. Compared to the monomer MLs, the unit size of the 2DPs is enlarged after the [4+4]-photopolymerization. The distance of the newly formed C-C bonds (σ-bonds) in the [4+4]-dimers is ca. 1.638 Å and 1.641 Å within 2DP **1** and **2** MLs, respectively.

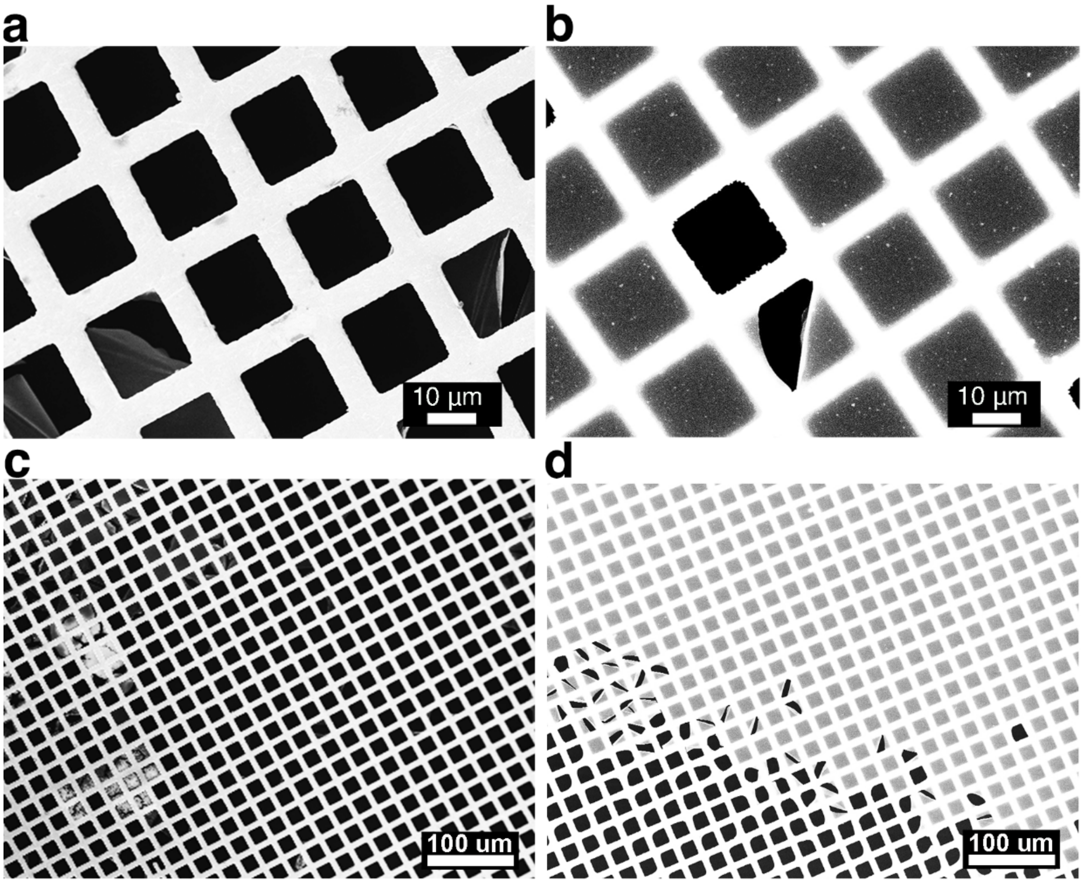

Supplementary Figure 7 | SEM images of monomer (a) **1** and (c) **2** MLs, and corresponding UV-driven 2DP (b) **1** and (d) **2** MLs suspended over Cu grids, respectively.

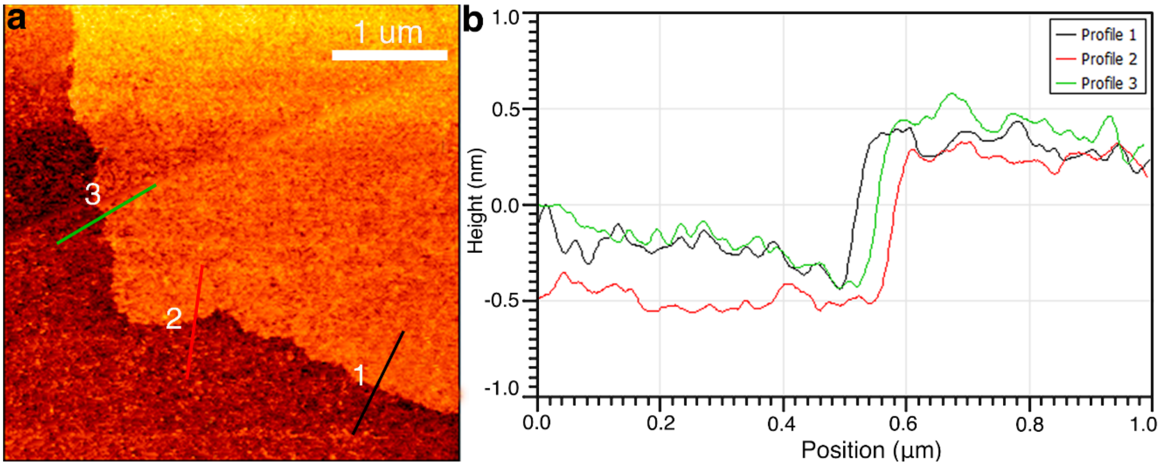

Supplementary Figure 8 | (a) AFM height image of monomer **1** ML on a SiO_2/Si substrate. (b) Corresponding line profiles along the color lines in (a).

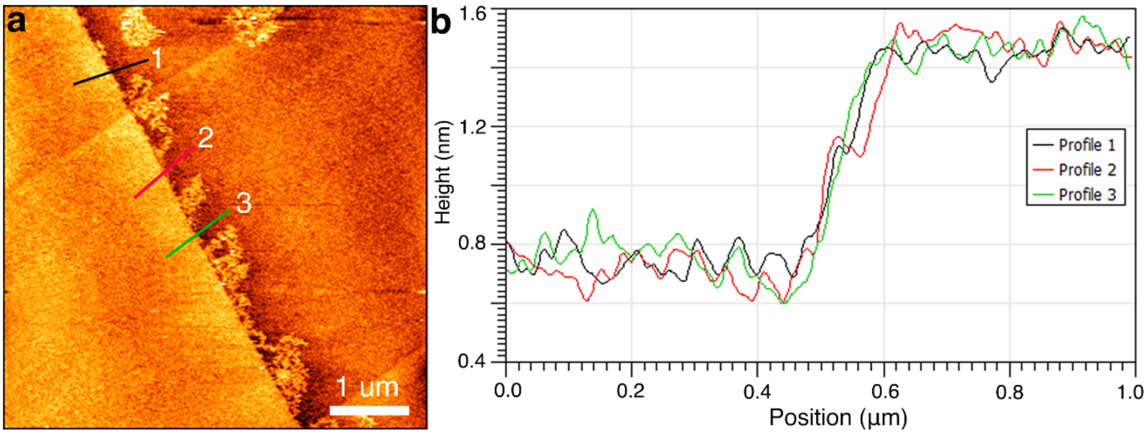

Supplementary Figure 9 | (a) AFM height image of 2DP **1** ML on a SiO_2/Si substrate. (b) Corresponding line profiles along the color lines in (a).

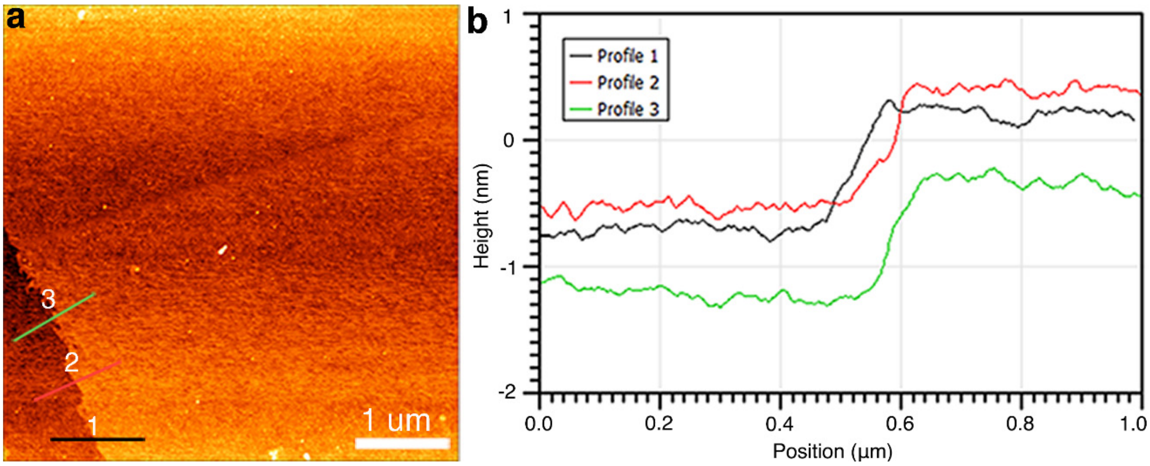

Supplementary Figure 10 | (a) AFM height image of monomer **2** ML on a SiO_2/Si substrate. (b) Corresponding line profiles along the color lines in (a).

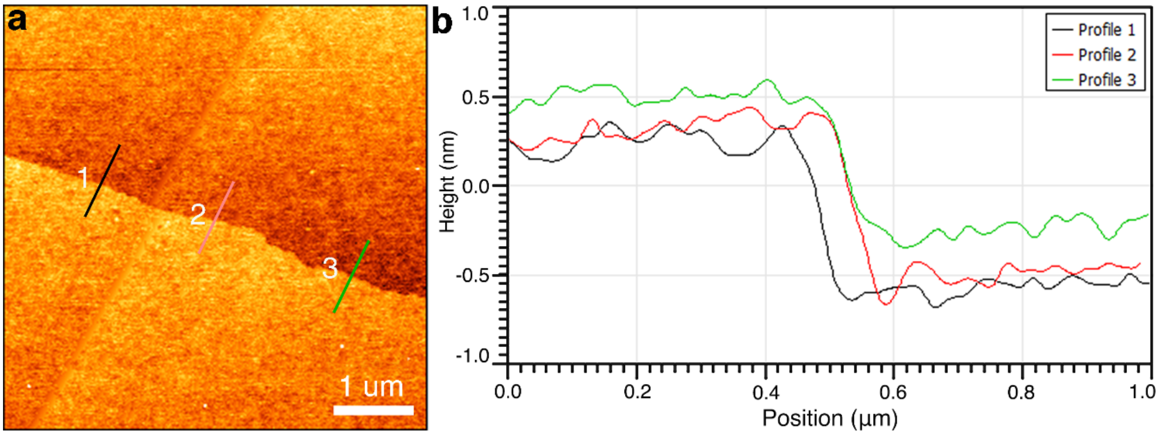

Supplementary Figure 11 | (a) AFM height image of 2DP **2** ML on SiO_2/Si substrate. (b) Corresponding line profiles along the color lines in (a).

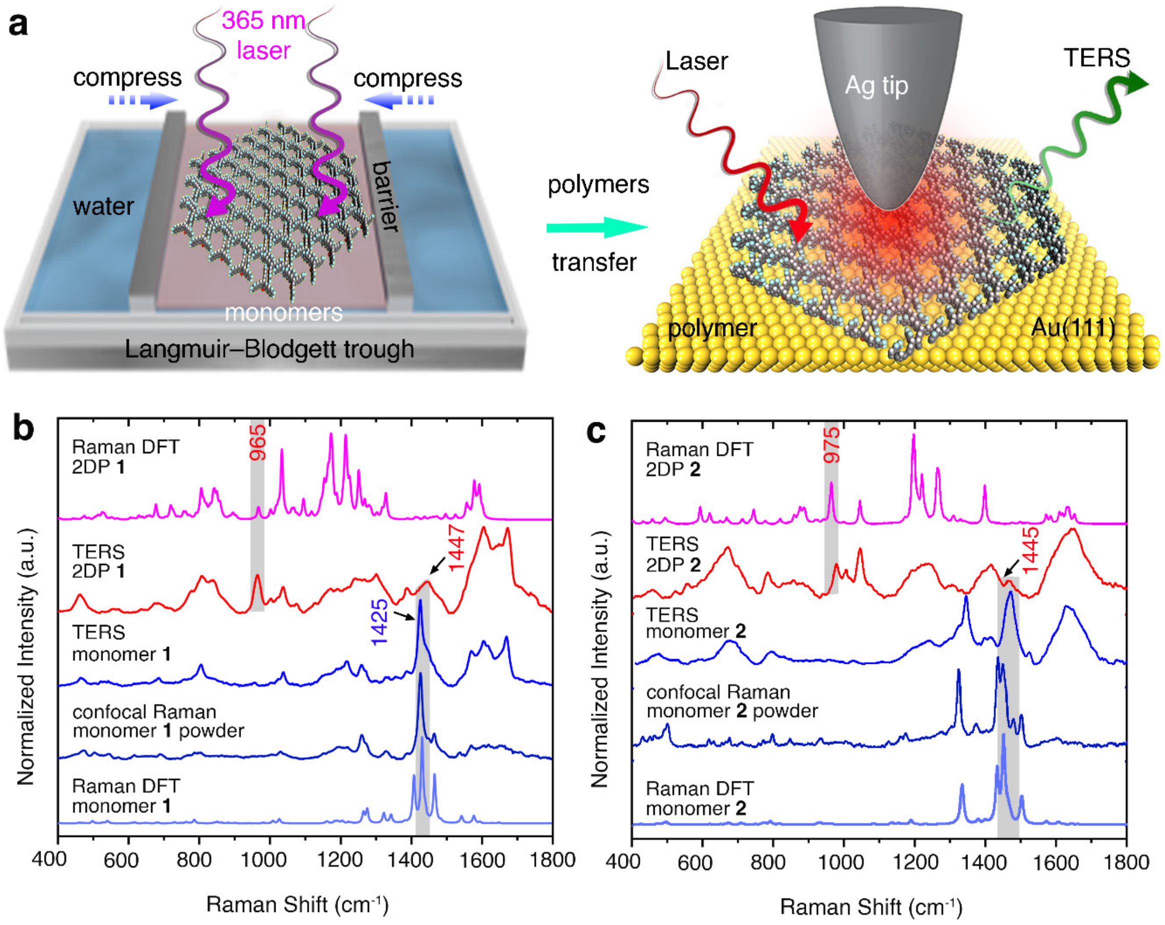

Supplementary Figure 12 | (a) Schematic illustration of the preparation of 2DP MLs at an air/water interface on a Langmuir-Blodgett trough with 365 nm laser irradiation for one hour. The monomer/2D polymer MLs can be transferred onto Au(111) by the Schäfer technique and then be characterized by TERS. (b) Typical experimental and calculated Raman/TERS spectra of powders/MLs of monomer **1** and MLs of UV-driven 2DP **1**. (c) Typical experimental and calculated Raman/TERS spectra of powders/MLs of monomer **2** and MLs of UV-driven 2DP **2**. The vibrational modes of representative bands at 965 cm^{-1} and 1425 cm^{-1} are labeled. a.u., arbitrary units.

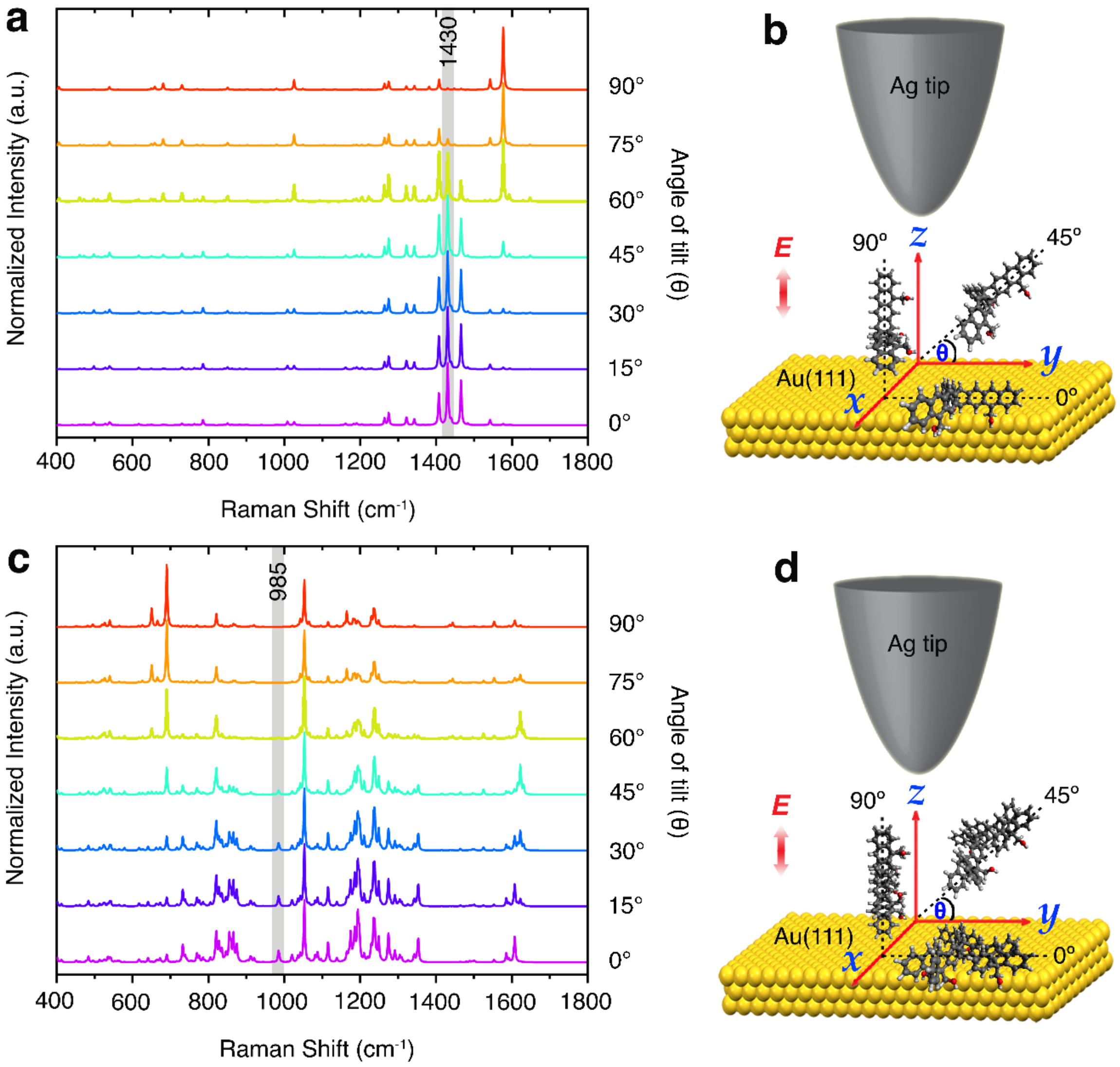

Supplementary Figure 13 | Calculated TERS spectra of (a) monomer **1** and (c) 2DP **1** models with different molecular orientations on Au(111). The calculated Raman intensity is normalized to the highest peak. (b, d) Schematics of the plane-parallel and plane-perpendicular orientations, respectively. Only the vertical compound of the local field E is considered for field enhancements. The molecular bending and twisting angles are held constant at 0°. a.u., arbitrary units.

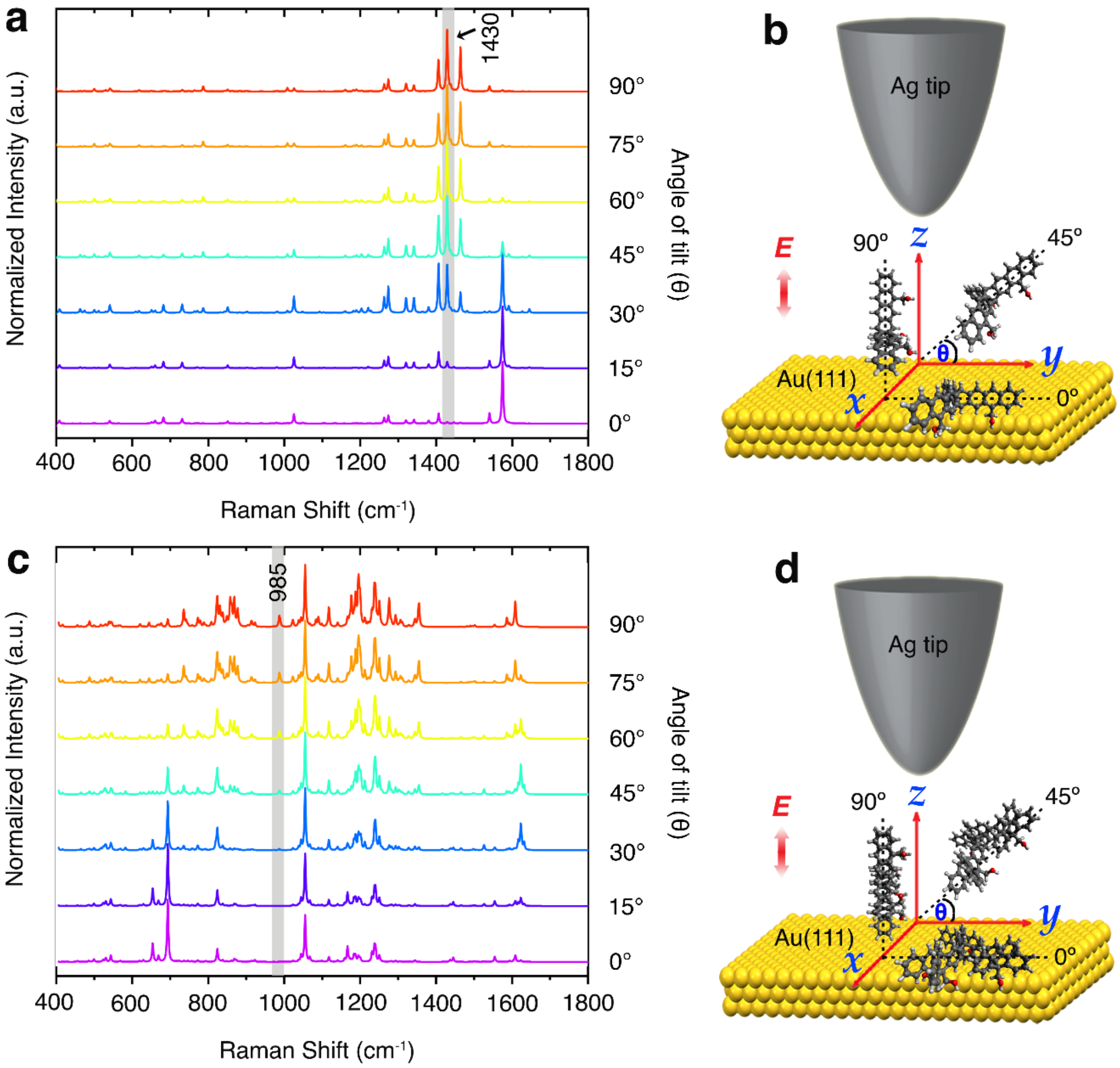

Supplementary Figure 14 | Calculated TERS spectra of (a) monomer **2** and (c) 2DP **2** models with different molecular orientations on Au(111). The calculated Raman intensity is normalized to the highest peak. (b, d) Schematics of the plane-parallel and plane-perpendicular orientations, respectively. Only the vertical compound of the local field E is considered for field enhancements. The molecular bending and twisting angles are held constant at 0°. a.u., arbitrary units.

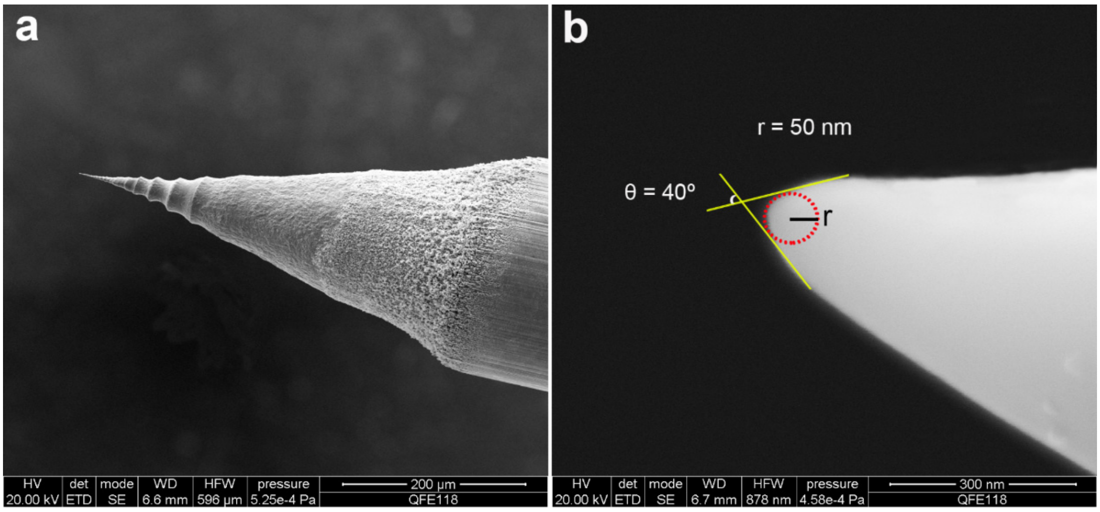

Supplementary Figure 15 | (a) SEM image of a typical Ag tip used in the STM-TERS study. (b) Higher magnification SEM image of the Ag tip. The curvature radius (r =50 nm) and the cone angle (θ = 40°) estimated from the SEM image were used for the FDTD models and field distribution calculations, as shown below in Supplementary Figs. 16-19.

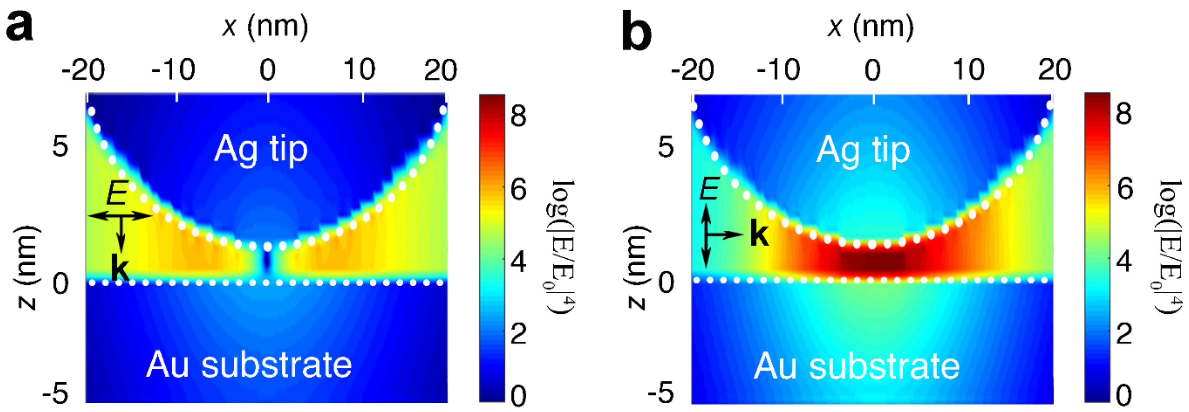

Supplementary Figure 16 | (a, b) FDTD simulated spatial distribution of the electric field at the Ag tip/Au substrate nanogap under 633 nm laser irradiation. E and E_0 denote the localized electric field and the incident electric field, respectively. k represents the wavevector of the incident light.

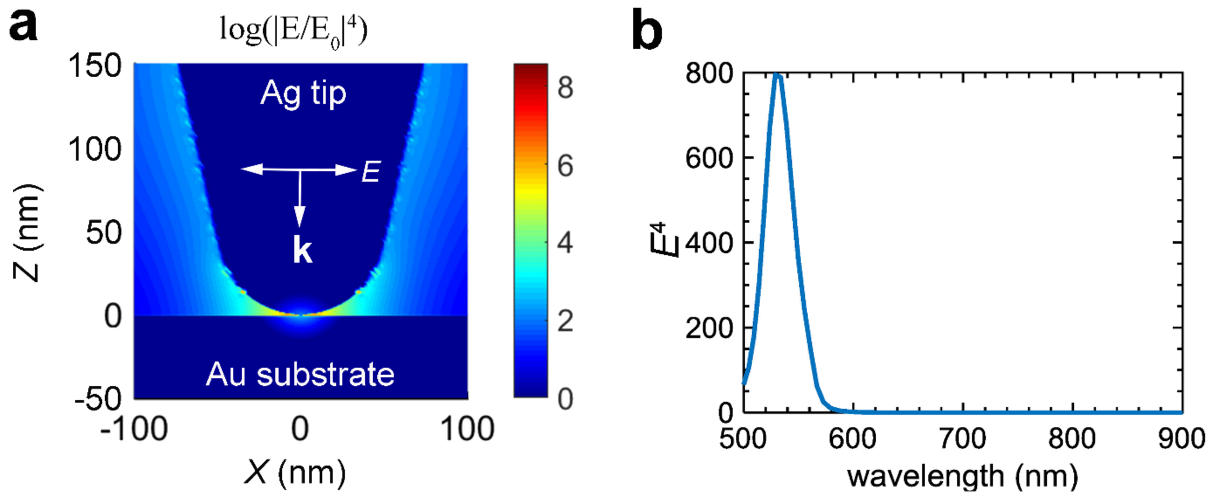

Supplementary Figure 17 | (a) Electric field distribution at the Ag tip/Au substrate 1-nm nanogap illuminated with 633 nm light along the tip axis. E and E_0 denote the localized electric field and the incident electric field, respectively. k represents the wavevector of the incident light. (b) Calculated electric field intensity at the Ag tip/Au substrate 1-nm nanogap with a vertical incident under different wavelength light irradiations. The simulated point is z = 0.1 nm above the Au substrate surface where z = 0 nm.

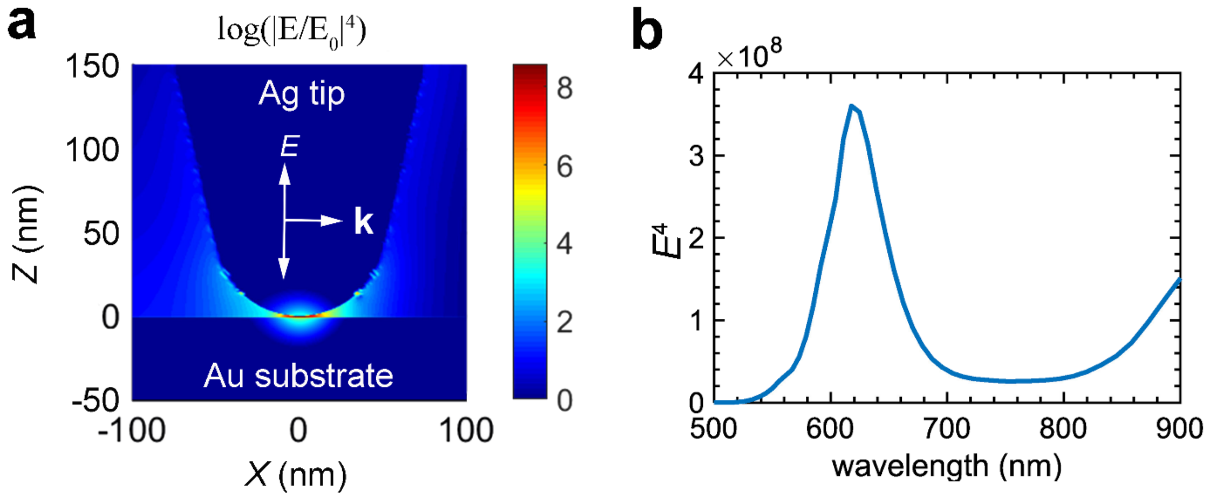

Supplementary Figure 18 | (a) Electric field distribution at the Ag tip/Au substrate 1-nm nanogap illuminated with 633 nm light along the substrate. E and E_0 denote the localized electric field and the incident electric field, respectively. k represents the wavevector of the incident light. (b) Calculated electric field intensity at the Ag tip/Au substrate 1-nm nanogap with a horizontal incident under different wavelength light irradiations. The simulated point is z = 0.1 nm above the Au substrate surface where z = 0 nm.

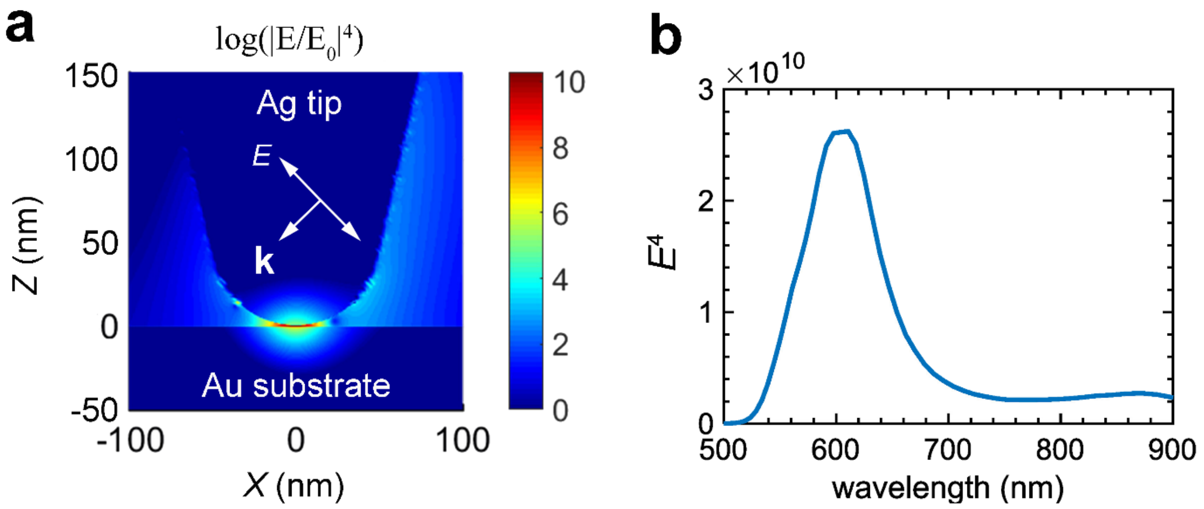

Supplementary Figure 19 | (a) Electric field distribution at the Ag tip/Au substrate 1-nm nanogap illuminated with 633 nm light tilted 45° with respect to the substrate. E and E_0 denote the localized electric field and the incident electric field, respectively. k represents the wavevector of the incident light. (b) Calculated electric field intensity at the Ag tip/Au substrate 1-nm nanogap with a tilted incident under different wavelength light irradiations. The simulated point is z = 0.1 nm above the Au substrate surface where z = 0 nm.

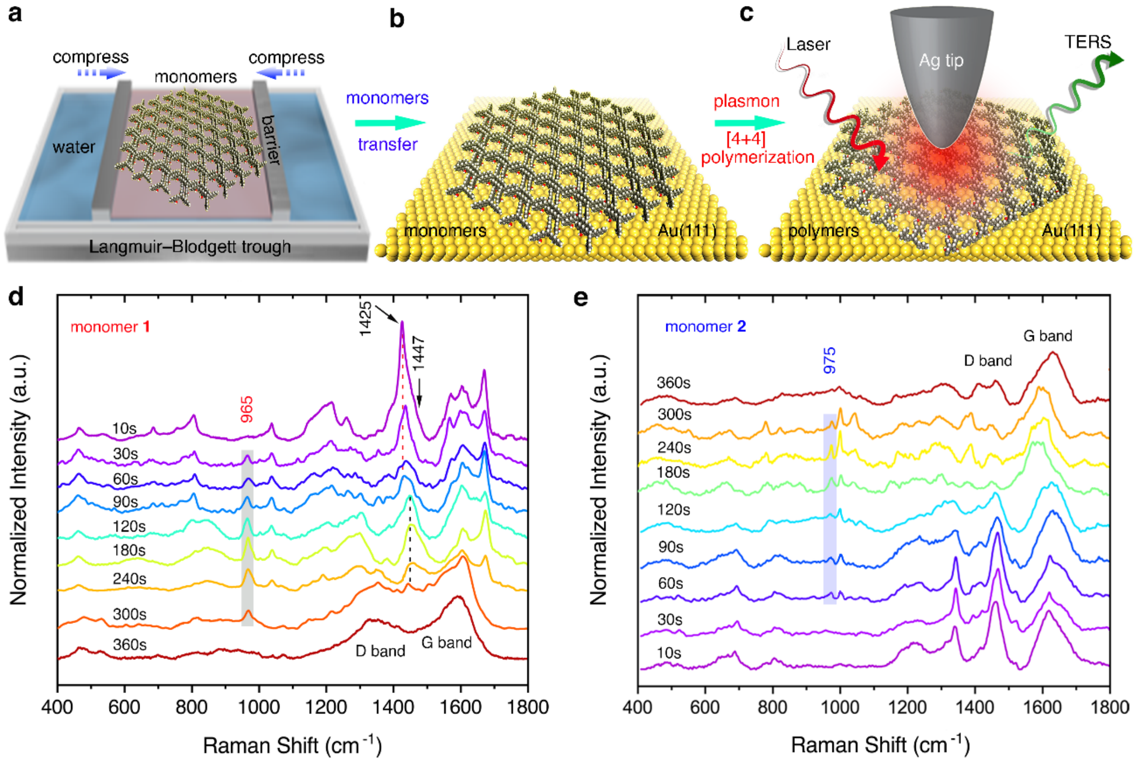

Supplementary Figure 20 | (a) Schematic illustration of the preparation of the monomer MLs at an air/water interface on a Langmuir-Blodgett trough. (b) Schematic illustration of the resulting monomer MLs on Au(111) transferred by the Schäfer technique. (c) Schematic illustration of the TERS experiments to trigger and monitor the plasmon-induced [4+4]-polymerization irradiated with 633 nm laser. (d, e) Time-dependent TERS spectral evolution of the monomer MLs in the STM-TERS nanogap. The TERS spectra were obtained with 633 nm laser excitation (0.3 mW) by positioning the STM Ag tip on the monomer MLs (0.1V, 0.1 nA). Serial TERS spectra were acquired with an integration time of every 10 s (see Figs. 2a, b in the main text). a.u., arbitrary units.

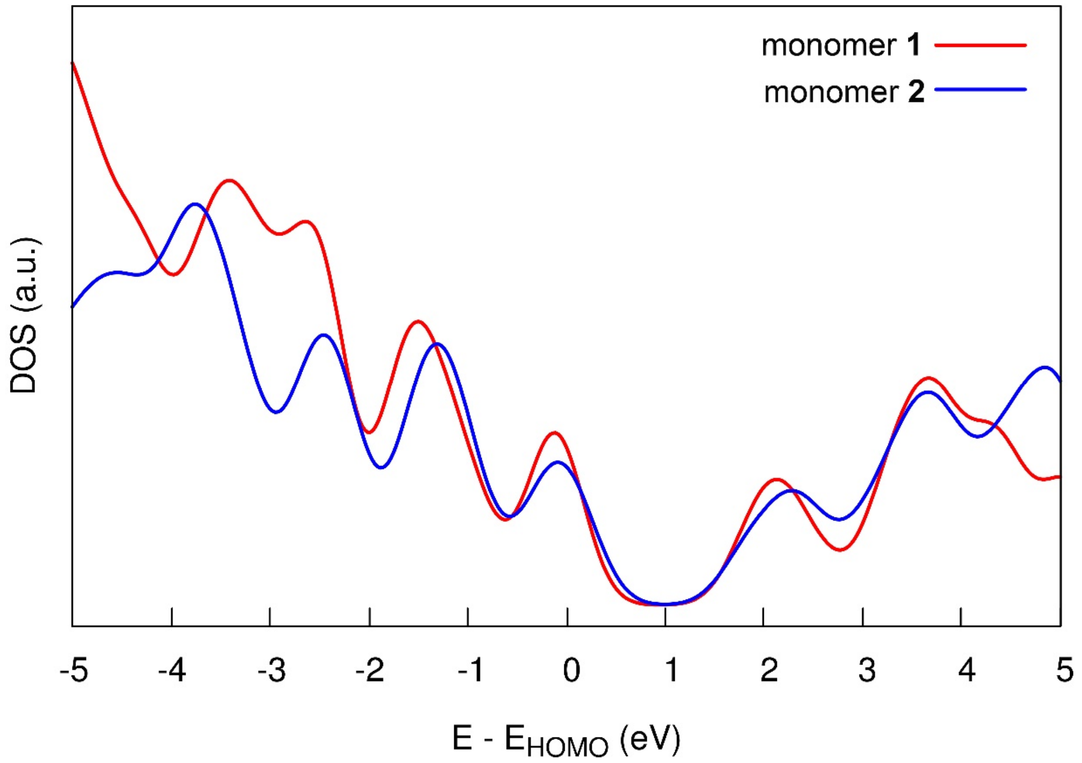

Supplementary Figure 21 | The calculated projected DOSs of monomer **1** and **2** MLs in a vacuum. Their packing configurations are shown in Supplementary Figure 4. The energy gap between the HOMO and the LUMO levels is approximately 2.26 eV and 2.41 eV for monomer **1** and **2** MLs, respectively. The molecules with lower HOMO-LUMO gaps can be more reactive since they can be excited by smaller energies. a.u., arbitrary units.

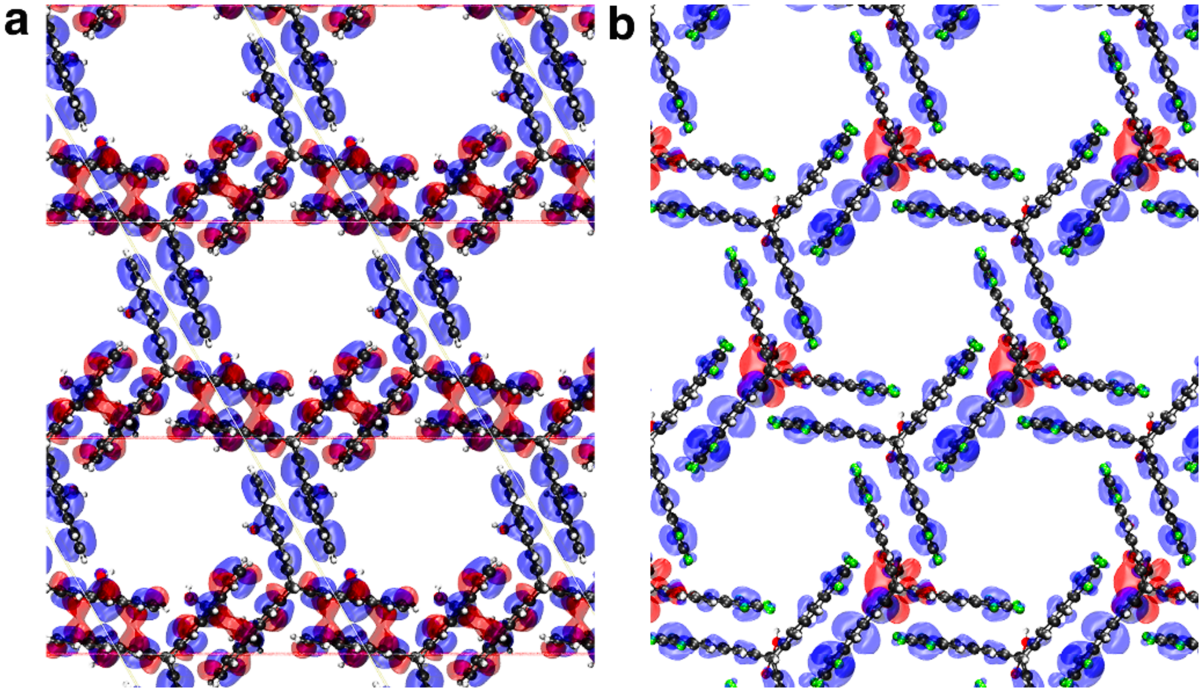

Supplementary Figure 22 | The spatial distribution of the (blue) HOMO and (red) LUMO levels for monomer (a) **1** and (b) **2** MLs in a vacuum. Spatial distributions of HOMO and LUMO are related to charge densities in frontier molecular orbitals, indicating that those constituent atoms in a molecule will serve as preferred sites for nucleophilic or electrophilic interactions.

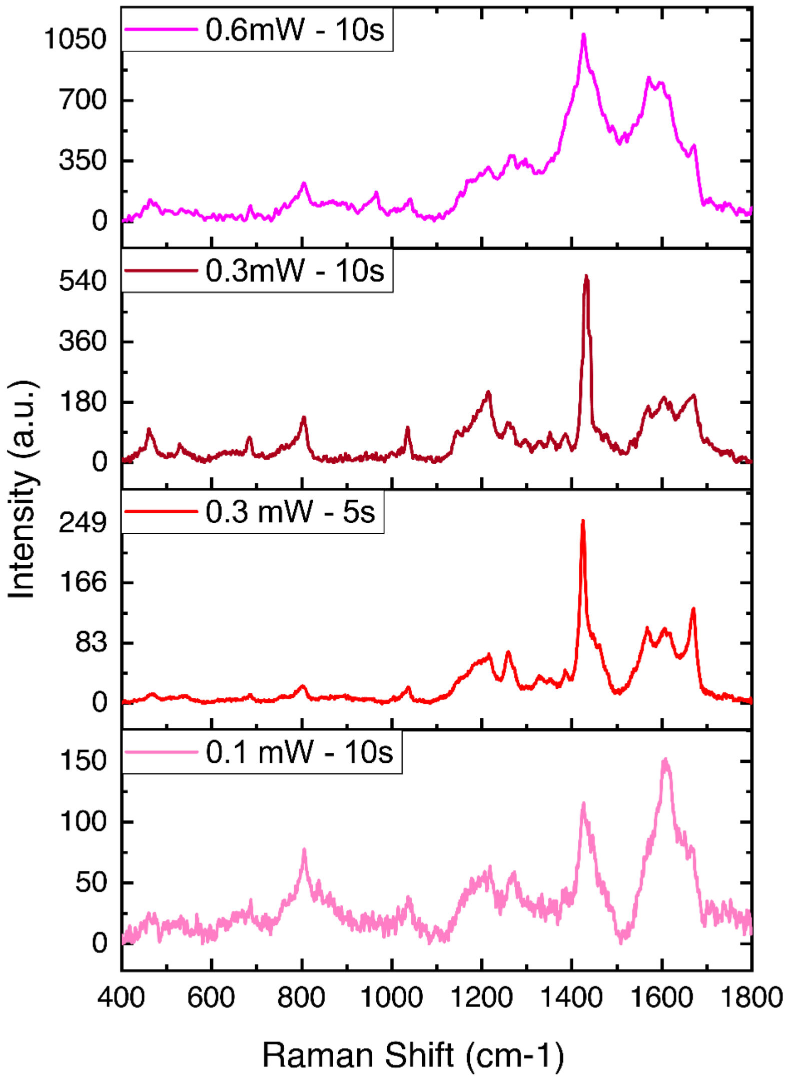

Supplementary Figure 23 | TERS responses of monomer **1** ML under 633 nm laser irradiation using different excitation powers and acquisition times. In order to obtain optimal spectra with good signal to noise ratios and avoid photodamages, the irradiation by 633 nm laser line with 0.3 mW incident power and 10 s acquisition time was applied for long-term TERS imaging. a.u., arbitrary units.

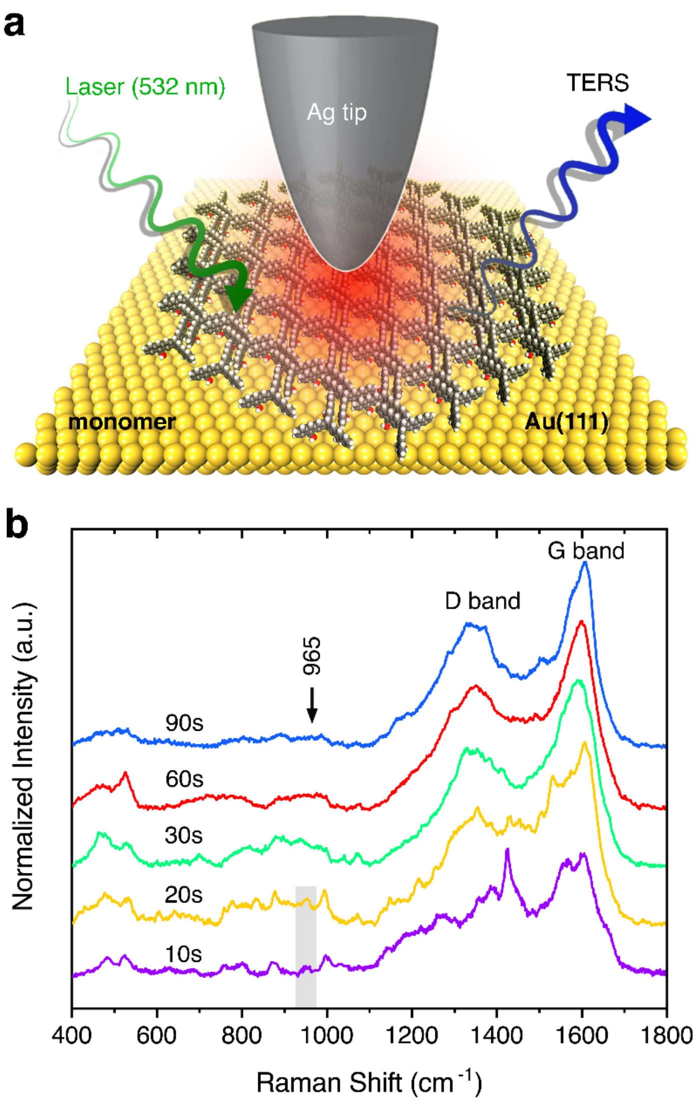

Supplementary Figure 24 | Schematic illustration of the TERS experiments to trigger and monitor the plasmon-induced [4+4]-polymerization under 532 nm laser irradiation. (b) Time-dependent TERS spectral evolution of monomer **1** ML in the STM-TERS nanogap (0.1V, 0.1 nA) under laser excitation (532 nm, 1.1 mW). Serial TERS spectra were acquired with an integration time of every 10 s. The 2D polymerization can also occur as indicated by the appearance of the 965 cm^{-1} band. Due to the lower resonance efficiency between 532 nm laser and the plasmonic nanogap, a higher incident power (1.1 mW) is required to get reasonable TERS signals. Thereby, plasmon-driven photodamages become more apparent in the hotspot under 532 nm laser irradiation. a.u., arbitrary units.

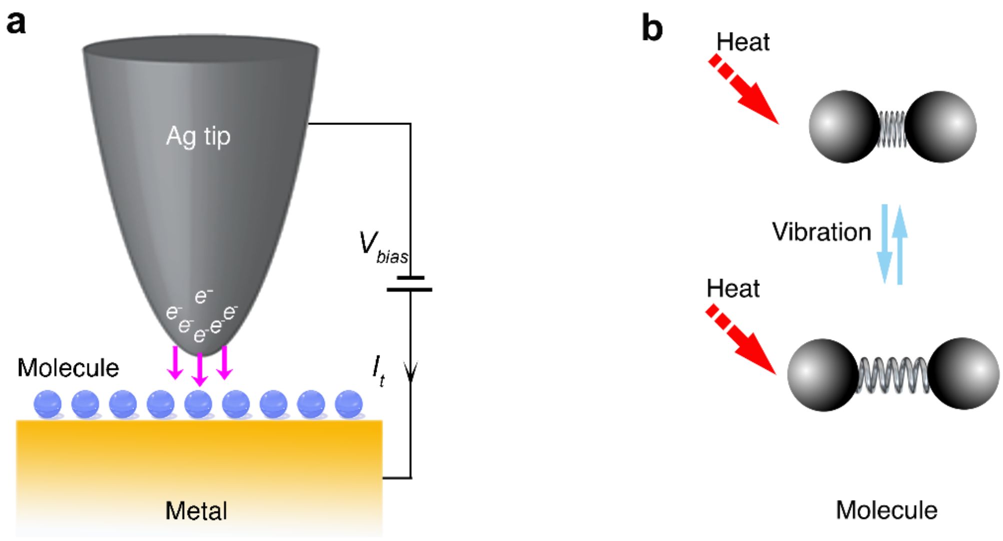

Supplementary Figure 25 | (a) Tunnelling electron injection stimulated by applied bias voltages. The tunneling electrons have an energy of $E_F \pm eV_{bias}$. (b) Vibrational excitation by the local heat caused by the decay of the local surface plasmon (LSP).

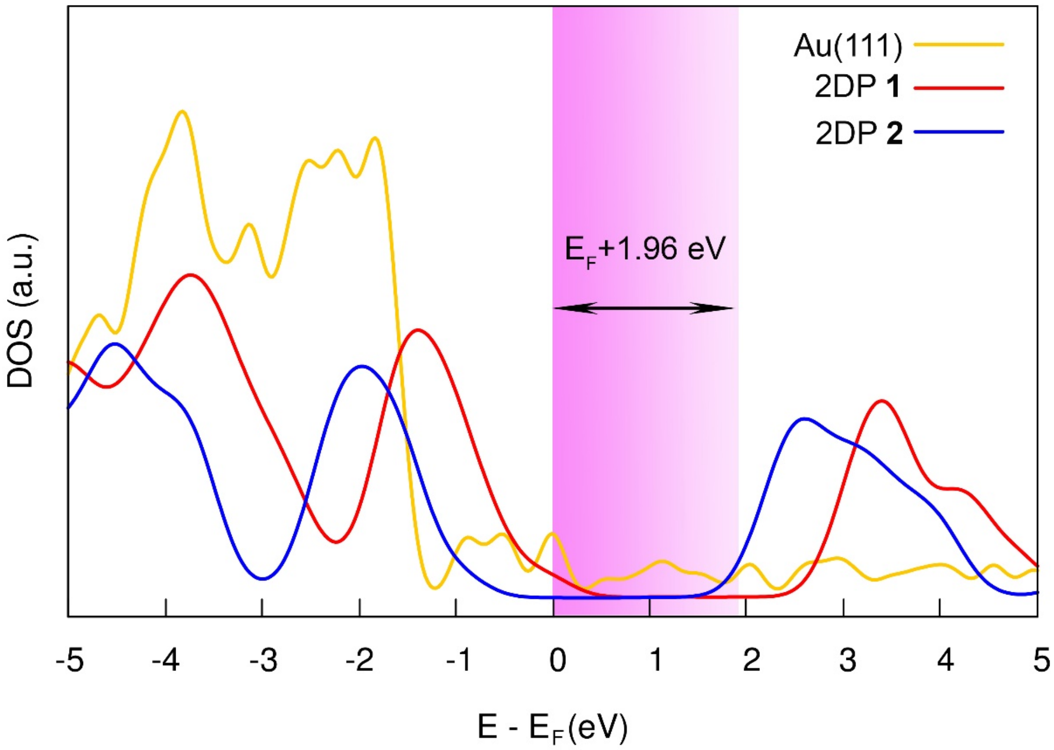

Supplementary Figure 26 | The calculated projected DOSs of 2DP **1** and **2** MLs on Au(111). The LUMO level is approximately 3.36 eV and 2.38 eV for 2DP **1** and **2** MLs, respectively. The highlighting belt indicates the energy distribution of the induced hot electrons in the TERS hotspot under 633 nm laser irradiation. a.u., arbitrary units.

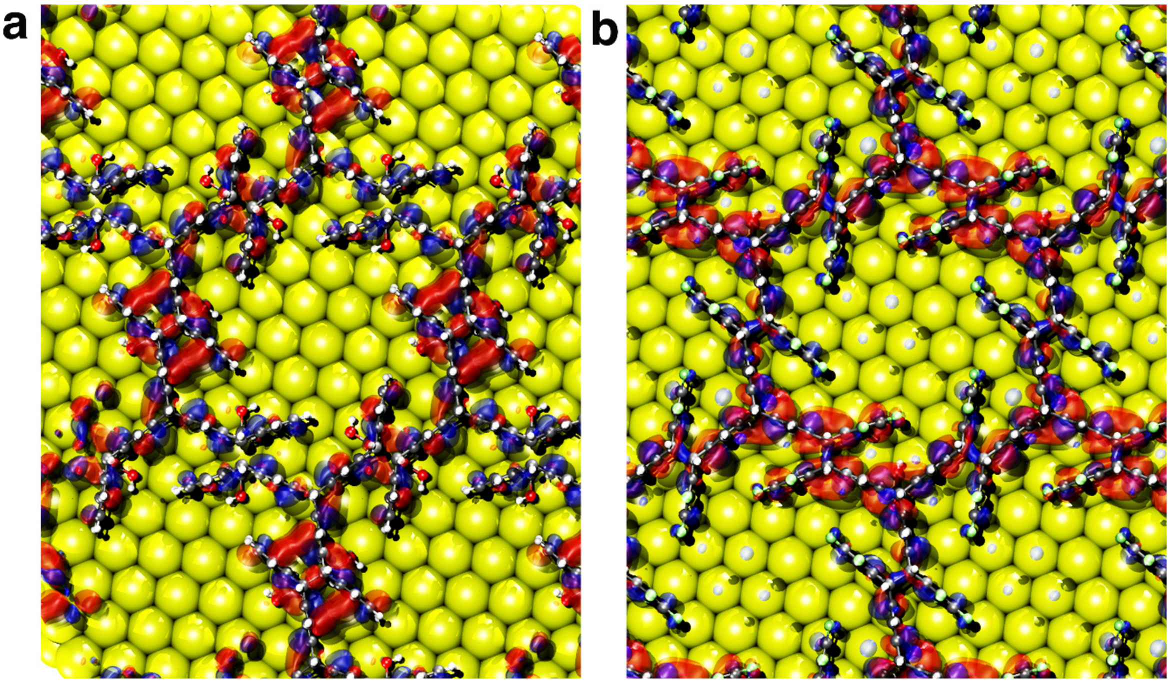

Supplementary Figure 27 | The spatial distribution of HOMO (blue) and LUMO (red) levels for 2DP (a) **1** and (b) **2** MLs on Au(111), respectively.

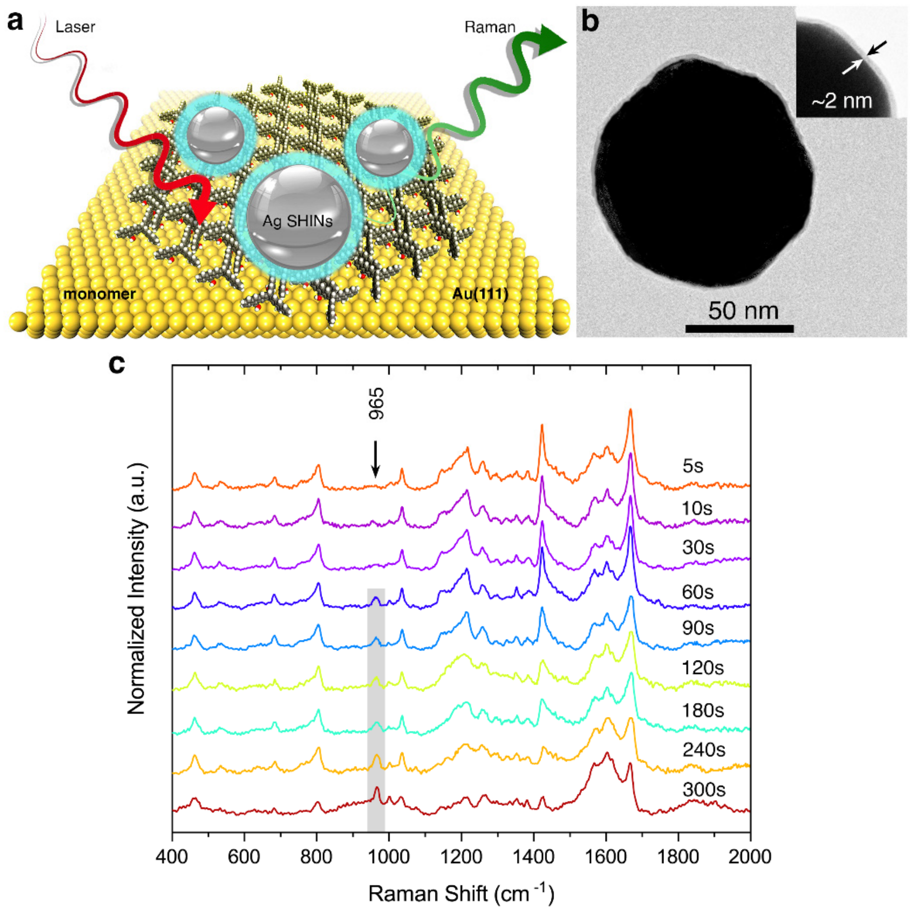

Supplementary Figure 28 | (a) Schematic illustration of in-situ SHINERS to monitor the [4+4]-cycloaddition reaction within monomer **1** ML on Au(111). (b) TEM images of a typical Ag@SiO_2 nanoparticle. Each shell-isolated nanoparticle (SHIN) is made up of a ~100 nm-sized Ag nanoparticle core covered with a ~2 nm thin pinhole-free SiO_2 shell. (c) Time-dependent SHINERS spectral evolution of monomer **1** ML on Au(111) under 633 nm laser excitation (5 s integration, 0.3 mW). The laser kept irradiation and spectra recorded at every 5 s. The plasmon-induced 2D polymerization can also be observed in this system after 60 s continuous laser excitation. Compared to the TERS system, this Ag@SiO_2/Au substrate configuration shows a lower efficiency for the hot carrier creation, which is probably due to the weaker plasmon resonance between the laser and the nanogap, caused by the larger size of the Ag nanoparticle and the bigger gap of the nanoparticle/substrate nanocavity than that in the TERS system.[16] a.u., arbitrary units.

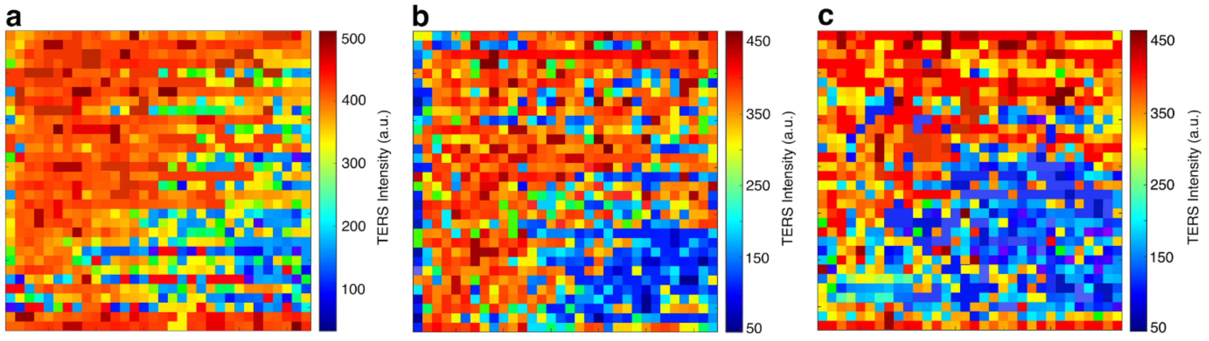

Supplementary Figure 29 | (a) The 1st, (b) 2nd, and (c) 3rd TERS intensity images at the 1425 cm^{-1} band. The maps were repeatedly measured over the 100 × 100 nm^2 area with 3.125 × 3.125 nm^2 pixels resolution at the same location under continuous laser irradiation (633 nm, 0.3 mW, 10 s integration). They are the complementary intensity maps to those in Figs. 5a-c (see the main text). a.u., arbitrary units.

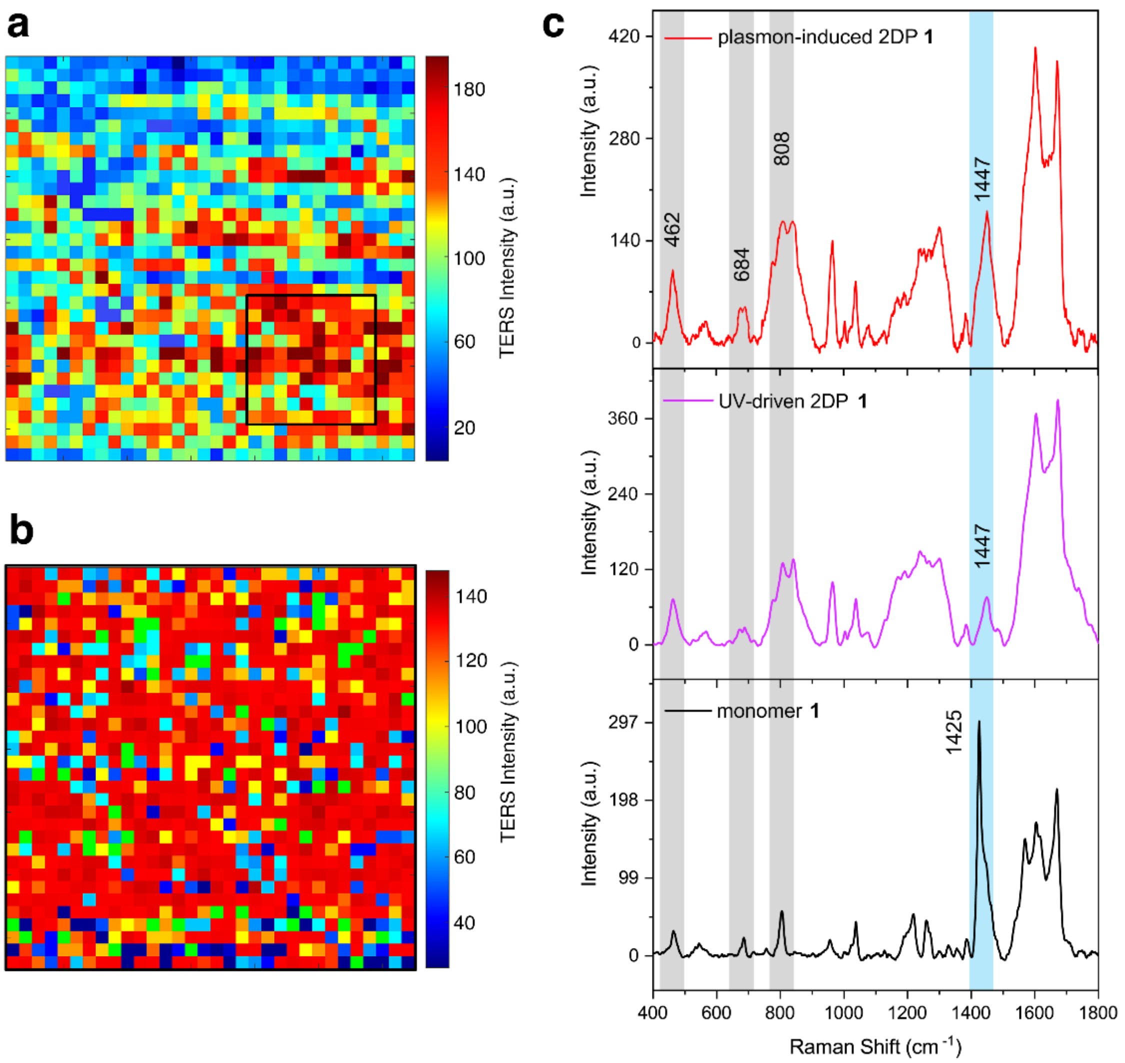

Supplementary Figure 30 | TERS intensity images at the 965 cm^{-1} band of (a) plasmon-induced and (b) UV-driven 2DP **1** MLs. The maps were measured over a 100 × 100 nm^2 area with 3.125 × 3.125 nm^2 pixels resolution under continuous laser irradiation (633 nm, 0.3 mW, 10 s integration). (c) Baseline corrected average TERS spectra of monomer **1** and corresponding 2DP **1** from corresponding TERS maps. The average spectrum of plasmon-induced 2DP **1** was averaged from 10×10 pixels (100 spectra, 31.25 × 31.25 nm^2) as selected by the black frame in (a, see Fig. 5c in main text). The average spectrum of UV-driven 2DP **1** was averaged from 32×32 pixels (1024 spectra, 100 × 100 nm^2). The average spectrum of monomer **1** was averaged from 32×32 pixels (1024 spectra, 100 × 100 nm^2, see Fig. 5a in main text). Spline baseline corrections were performed in Origin 9.1 software by the 2nd derivative method with 30 anchor points. The detailed calculation of conversion numbers and the standard errors are shown below in Table S1. a.u., arbitrary units.

Supplementary Table 1. Estimation of polymerization conversions of UV-driven and plasmon-induced 2DP **1**. All peak intensity/ratio data are collected from TERS maps with 32×32 (the UV-driven sample, see Supplementary Figure 29) and 10×10 (the plasmon-induced sample, see Supplementary Figure 29) pixels, and the values are given in the format of mean(M) ± standard error (SE, δ).[1]

**Peak** / **Intensity (a.u.)**	462 (cm^{-1})	684 (cm^{-1})	808 (cm^{-1})	1425/1447 (cm^{-1})
monomer **1**	32.412±0.184	23.145±0.146	53.362±0.364	298.453±1.885
Peak Ratios (I_{1425}/I_{peaks})	9.216±0.025	12.904±0.041	5.592±0.021	
UV-driven 2DP **1**	72.432±0.484	37.231±0.224	129.325±0.962	74.243±0.516
Peak Ratios (I_{peaks}/I_{1447})	1.023±0.005	1.994±0.006	0.575±0.002	
Conversion	88.9%±1.6%	84.5%±1.5%	89.7%±2.1%	average 87.7%±1.8%
Plasmon-induced 2DP **1**	92.258±0.752	59.841±0.328	155.468±1.987	168.375±2.184
Peak Ratios (I_{peaks}/I_{1447})	1.717±0.008	3.026±0.014	1.083±0.006	
Conversion	81.4%±2.1%	78.2%±2.3%	80.6%±2.6%	average 80.1%±2.3%

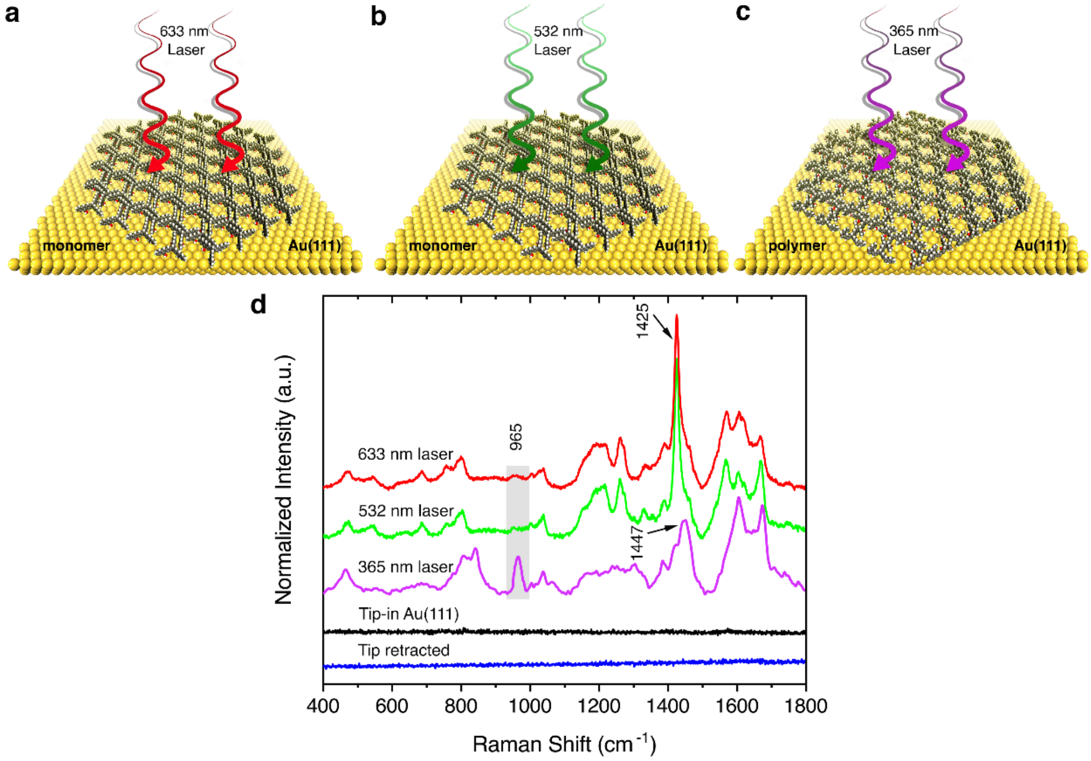

Supplementary Figure 31 | Schematic illustration of monomer **1** MLs on Au(111) under (a) 633 nm, (b) 532 nm, and (c) 365 nm lasers illumination for one hour. Their incident powers were adjusted to a similar level (~2.5 mW). The face-to-face stacking MLs were obtained at an air/water interface and then transferred onto Au(111) by the Schäfer technique. (d) Early-stage TERS spectra of the MLs on Au(111) after irradiating by the above lasers. The TERS spectra were recorded from the MLs excited with the 633 nm laser (0.3 mW, 10 s integration). After the intensive UV laser (365 nm) illumination on monomer **1** ML, the early-stage TERS spectrum suggests the occurrence of the UV-driven [4+4]-photopolymerization on Au(111), indicated by the appearance of the 965 cm^{-1} band.[17] However, the reaction cannot be triggered by 633 nm or 532 nm laser illumination without generating the resonant plasmon and the transfer of the induced hot carriers. a.u., arbitrary units.

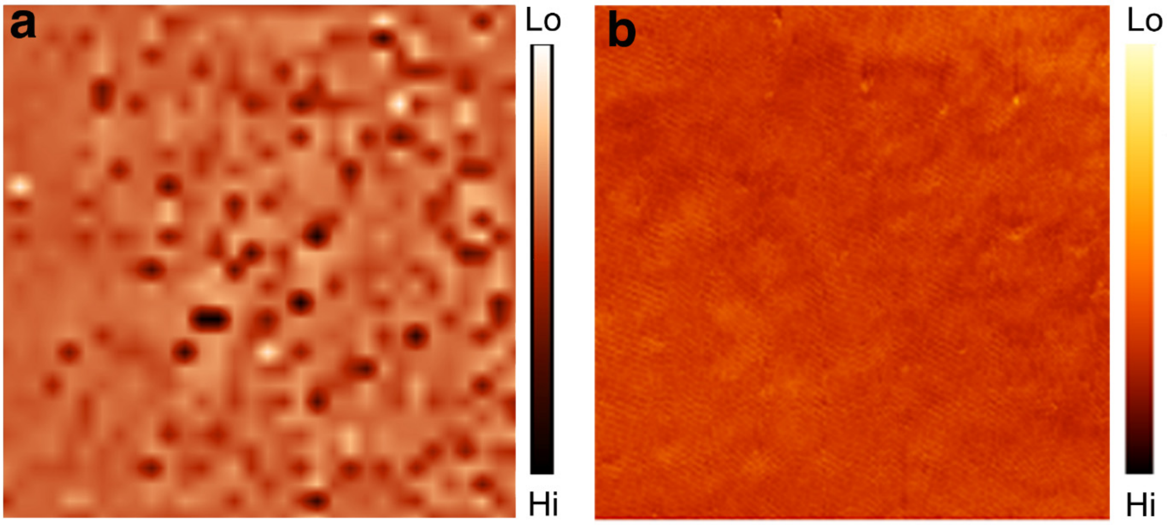

Supplementary Figure 32 | (a) The low-resolution STM image (32 × 32 pixels) simultaneously acquired during the TERS imaging (see the main text, Fig. 4) over a 100 × 100 nm^2 area (0.1 V, 0.1 nA). (b) The higher resolution STM image (512 × 512 pixels) was recorded after TERS imaging in the same area (0.1 V, 1.0 nA). Due to the limited stability of the TERS setup under ambient conditions, it is virtually impossible to obtain atomic STM imaging of monomer **1** MLs prepared by the LB trough.

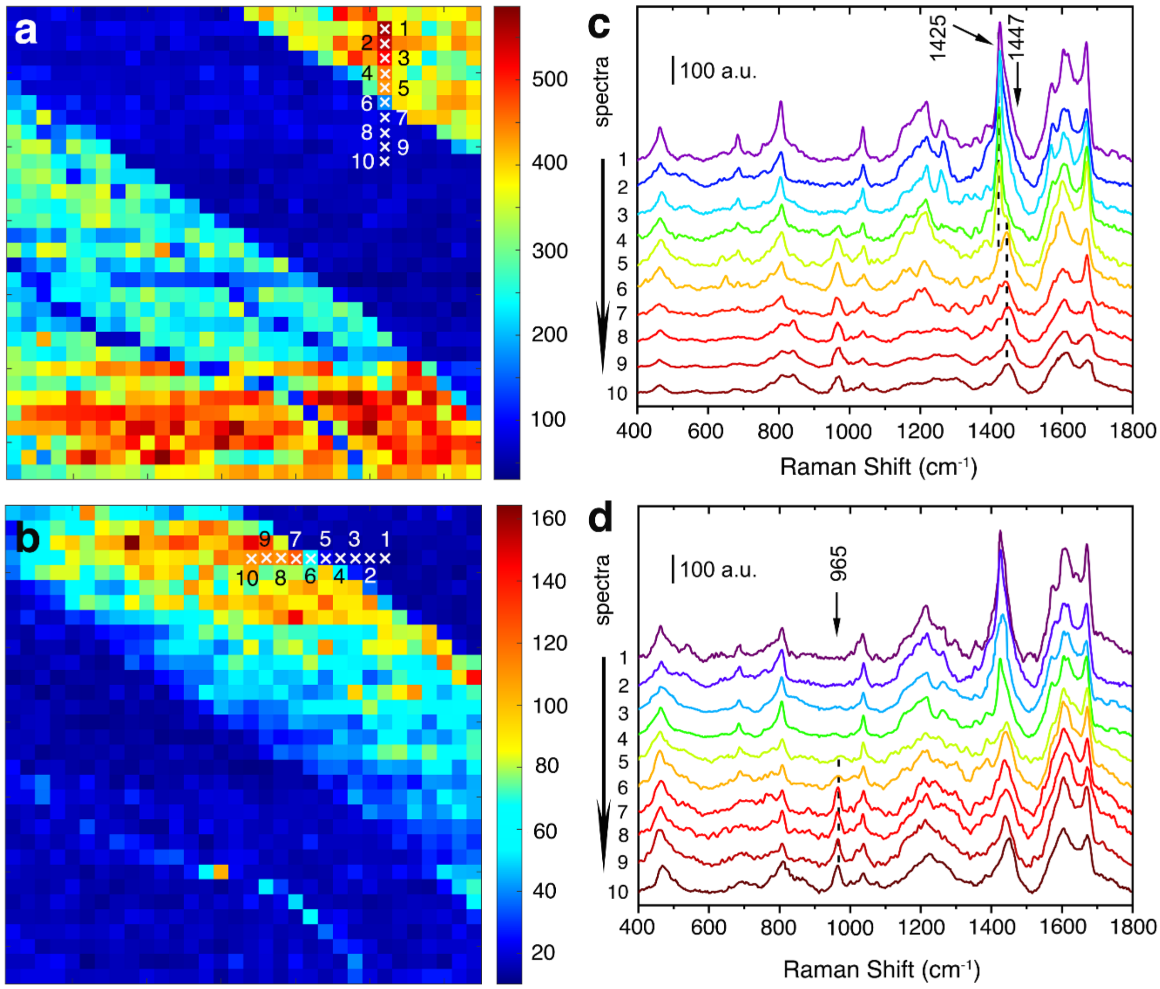

Supplementary Figure 33 | Complementary TERS intensity images of the bands at (a) 965 cm^{-1} and (b) 1425 cm^{-1} of monomer **1** ML on Au(111). The maps were measured over a 100 × 100 nm^2 area with 32 × 32 pixels under continuous laser irradiation (633 nm, 0.6 mW, 10 s integration). (c, d) Line-trace TERS spectra acquired along the white star lines in (a) and (b), respectively. Distinct phase segregation between the monomer and polymer becomes visible at the nanoscale when sufficient peculiar hot carriers are generated. Such pattern formation is probably due to a different packing array of the monomer MLs or a different facet orientation on Au(111), and thus leading to the discrepancy in the plasmon-assisted catalytic activity.[18, 19] Note that the packing configuration of monomer arrays, the distance between two anthracene blades, the angle of π-π stacking modes, as well as the contact between the MLs and Au(111) could impact and tune the plasmon-induced [4+4]-polymerization, and thus affect the fabrication of localized nanopatterns.[20, 21] a.u., arbitrary units.

3. Supplementary Methods

3.1 Langmuir-Blodgett (LB) Trough and Monolayer (ML) Preparation

Amphiphilic monomers **1** and **2** were synthesized as described in our previous work.[1, 2] Briefly, Langmuir experiments were carried out on a KSV 2000 System 2 trough equipped with a platinum Wilhelmy plate and a dipper. The trough is made from Teflon and the barriers from Delrin. Prior to each experiment, the trough was cleaned with a sequence of millipore H_2O, $CHCl_3$, EtOH, millipore H_2O, wiped dry using dust-free papers (Kimtech Science Precision Wipes, Kimberly-Clark Professional, Woodbridge, Ontario, Canada) after each solvent, and given a final rinse with millipore H_2O. The barriers were cleaned with millipore H_2O, EtOH, and then again millipore H_2O wiped off with Kimtech Precision Wipes after each solvent and were given a final rinse with millipore H_2O. The stage carrying the substrate was immersed in the water prior to the experiment (transfer from below). 60-80 μL of monomer stock solution (0.5 mg/mL in $CHCl_3$) was applied using a 100 μL glass microsyringe. After delaying 30 min, which allowed for evaporation of the solvent, the barriers were compressed at a speed of 3 mm/min to a surface pressure of 20 mN/m. For BAM imaging, a KSV MicroBAM (KSV NIMA, Finland) was used, which operated with a 659 nm laser.

Photopolymerization on a trough was carried out using LEDs of wavelength λ = 365 nm (Omicron Laser LEDMOD365.250.OEM) after the ML film had been kept at a surface pressure of 20 mN/m for 1 h. The silicon wafers for transfer (Si/SiO_2, Thermo, 300 nm) were cleaned with Piranha solution (H_2SO_4/H_2O_2 3:1) under sonication for 15 min, followed by sonication in millipore H_2O (3 ×15 min). This removed a photoresist coating (10 μm of AZ9260, Merck Performance Materials GmbH, Germany). The wafers were then kept under millipore H_2O. Prior to use, they were rinsed with EtOH and then millipore water.

3.2 Langmuir-Blodgett (LB) Film Transfer and Spanning

Film transfer was achieved by raising the stage carrying the substrate at a lifting speed of 0.5 mm/min. The stages were either horizontal or had a tilt angle of 45°. In the case of film transfer onto TEM grids, the copper grids with a mesh size of 1000 (PLANO, G2780C) were gently placed from the top onto the film with tweezers. A piece of writing paper was then placed onto the grids and, upon adsorption of the grids to the paper, peeled off the water surface and dried.

3.3 Scanning Electron Microscopy (SEM)

The TEM grids were placed on a holder (PLANO, G3662) and imaged with FEG-SEM (Zeiss LEO Gemini 1530, Germany) microscope with an in-lens detector.

3.4 Atomic Force Microscopy (AFM)

Tapping mode AFM height analysis and imaging were carried out on a Bruker Dimension Icon (Bruker Corporation, USA) using OMCL-AC160TS silicon tips (Asylum Research Cypher S, Oxford Instruments, UK) with a resonance frequency between 200 and 400 kHz and a spring constant of about 42 N/m.

3.5 UV-Vis Spectroscopy

UV-Vis spectra of monomer **1** in acetonitrile and the corresponding LB monolayer on quartz were recorded by a UV-Vis spectrophotometer (UV-1800, Shimadzu, Japan). The scanning speed was 400 nm/min with a spectral resolution of 1 nm. The substrates used were Suprasil quartz (Suprasil 1, 0.5 mm thick, Heraeus, Hanau, Germany).

3.6 Raman and TERS measurements

All TERS and confocal Raman spectra were acquired on a combined STM/Raman microscope (Ntegra Spectra, NT-MDT, Zelenograd, Russia) enclosed by a home-made acoustic isolation box and operated under ambient conditions. The instrument is equipped with an air objective (100 ×, NA = 0.7, Mitutoyo, Japan) and an electron-multiplying charge-coupled device (EMCCD, Newton 971 UVB, Andor, Belfast, UK). The spectrometer was calibrated by a standard neon lamp (Renishaw, UK). TERS probes were prepared by electrochemical etching of a silver wire (diameter 0.25 mm, 99.9985% purity, Alfa Aesar). A 3:1 (v/v) mixture of ethanol (absolute, Sigma-Aldrich) and perchloric acid (70%, Sigma-Aldrich) was used as the etchant. A potential of 8 V was applied, and a current of ≈10 mA was measured during the etching process. TERS maps were collected in STM feedback (constant current mode) with the sample surface be moved by the piezo sample stage in the *x*, *y*, and *z* directions while keeping the relative laser-to-tip position fixed. The dynamic range of the piezo scanner (6.0 μm × 6.0 μm in *xy* and 2.0 μm in *z* with 220 V) is small, which ensures highly precise control of the tip–substrate gap and *xy* coordinate with *a* < 0.1 nm step size. An exposure time of 5 s and 2 accumulations were used for all spectra. A 632.8 nm HeNe laser at an incident power of 0.3 mW was used as the excitation source. During TERS measurements, the bias voltage was set to 0.1 V, and the tunneling current was kept at 0.1 nA to avoid scratching the sheet. All TERS images were obtained after overnight measurements (continuous laser illumination and data recording) to minimize the thermal drift of the system. In order to get better STM imaging, the bias voltage at 0.1 V and tunneling current at 1.0 nA were applied after TERS mapping. Ag@SiO_2 shell-isolated nanoparticles (SHINs) were donated by Prof. Jian-Feng Li's group (Xiamen University, China), prepared according to their previous work.[22, 23]

3.7 FDTD Calculations

The spatial distribution and absorption spectra of a local electric field in the nanogap between the Ag tip and Au substrate were calculated using the FDTD method (Lumerical Solution). The simulation model comprised an electrochemically etched Ag tip (curvature radius r=50 nm, cone angle θ=40°, Supplementary Figure 15), an Au metal slab, and a vacuum layer. The tip–surface distance is set at 1 nm, and the incident wavelength set at 633 nm. The dielectric function of Au and Ag was taken from the experimental data reported by Johnson and Christy.[24] A plane wave, which was polarized along the *x*-direction and propagated along the *z*-direction, impinged on the nanogap with different incident angles (Supplementary Figs. 16-19). In order to obtain the absorption spectra, the incident angles fixed while changing the incident wavelengths (Supplementary Figs. 16-19).[25, 26]

3.8 DFT and TERS Calculations

Theoretical Raman spectra were calculated using the Gaussian 09 software (Gaussian, Wallingford, USA) by means of DFT. All calculations, including full geometry optimizations and frequency predictions, were performed using B3LYP/6-31+G(d) basis. The keyword Integral = (Grid=UltraFine, Acc2E=11) was used to increase the two-electron integral accuracy when SCF calculations failed to converge using default run parameters. All calculated frequencies were scaled with proper factors compared to confocal Raman spectra. The optimized geometry and the calculated vibrational modes were visualized using the Gaussview 5 package.

TERS calculations based on the dipole approximation were carried out to study molecular orientation dependence as described previously.[27, 28] The Raman responses are related to the magnitude of Raman tensor α and the intensity of the local electric field. When a molecule is

adsorbed to a flat metallic surface, the Raman signals change with the orientation of molecules because the Raman tensor α will be modified after taking surface selection rules into consideration. The intensity of gap-mode TERS signals can be approximately expressed as follows:[27]

$$I_{\mathrm{TERS}} \propto |\alpha_{\mathrm{ZZ}}|^2$$

The Raman tensor can be obtained from DFT calculations, expressed as X, Y, and Z components. Moreover, the excitation of gap-mode surface plasmon resonance is based on the interaction between the incident laser and the tip-substrate gap junction by lightning rod and antenna effects.[29] Under our STM-TERS configuration, the major local field enhancement is contributed by the vertical components (ZZ axis), which are much larger than the horizontal components (XY axis).[25] Additionally, the local field polarization vertical components (ZZ axis) are perpendicular to the metallic surface (XY axis). Together, combined with the surface selection rules, only the vertical components of the Raman tensor (α_{ZZ}) can be effectively enhanced by the vertical plasmon-enhanced field (ZZ axis) to provide primary TERS intensity. However, when a molecule is tilted up on the surface, the Raman tensor will change as well, depending on the tilt angle. Correspondingly, some horizontal components of the Raman tensors could contribute to a new effective nonzero α_{ZZ} components, while the previous α_{ZZ} components could be changed. Note that our TERS calculations are based on a simplified Raman tensor model, neglected intermolecular and molecule-substrate interactions.

The projected density of states (DOSs) and the HOMO/LUMO calculations were performed using the CP2K package.[30] PBE functional with Grimme D3 correction was used to describe the system.[31, 32] Kohn-Sham DFT has been used as the electronic structure method in the framework of the Gaussian and plane waves method.[33, 34] The Goedecker-Teter-Hutter (GTH) pseudopotentials,[35, 36] TZV2P-MOLOPT-SR-GTH basis sets were utilized to describe

molecules and DZVP-MOLOPT-SR-GTH for the gold (111) surface.[33] A plane-wave energy cut-off of 400 Ry has been employed. The Fermi-Dirac smearing has been applied to study the metallic system, and the electronic temperature is set to 300K. The gold surface containing 172 metal atoms was shaped to obtain (111) surfaces with four ($\sqrt{43} \times \sqrt{43}$) atomic layers within a hexagonal 19.34 × 19.34 × 30 $Å^3$ box. All the simulations are carried out by keeping the two bottom metal layers fixed at the initial coordinates in order to maintain the bulk behaviour of the inner part of the slab. The MLs are adsorbed on the metal surface with the OH/COOH group orientated to the surface.

The adsorption energy is defined as

$$E_{\mathrm{ad}} = E_{\mathrm{mol/sur}} - E_{\mathrm{mol}} - E_{\mathrm{sur}} \quad (1)$$

where E_{ad} is the adsorption energy, $E_{\mathrm{mol/sur}}$ is the total energy for a molecule on the surface, E_{mol} and E_{sur} are the energy of isolated molecule and surface.

3.9 Conversion Ratio Calculations

In order to estimate polymerization conversion, an internal standard method was applied for analysis, as described in our previous work.[1] For example, the intensive 1425 cm^{-1} band of monomer **1**, which majorly corresponds to ring breathing vibrations of the 9-(hydroxymethyl)anthracene blades (see Movie S1), was chosen as an indicator for this compound. After polymerization, the weak 1447 cm^{-1} band in the polymer suggests some residual monomer. The bands at 462, 684, and 808 cm^{-1}, which mostly correspond to vibrations of the center triptycene core, are basically unaffected by the polymerization and are therefore selected as internal references. After normalizing these signals for both monomer **1** (m) and 2DP **1** (p), conversions were obtained by determining the peak intensity ratios of the reference

vibrations with the signal at 1425/1447 cm^{-1}. The calculation details of conversion ratio for monomer **1** and 2DP **1** are shown below:

$$C_{\text{conversion ratio}} = 1 - \frac{M_\mathrm{p}}{M_\mathrm{m}} = 1 - \frac{\frac{I_{1425}}{I_{\text{references}}}}{\frac{I_{1447}}{I_{\text{references}}}} \quad (2)$$

where M_p is the mean value of the normalized peak ratio (for residual monomer signal) from polymer samples, and M_m is the mean value of normalized peak ratio (for original monomer signal) from monomer samples. In our measurements and calculations, M_p and M_m are independent variables.

3.10 Spectral Processing

For all experimental TERS imaging and spectra shown, the data were fed into MatLab (R2020a, MathWorks, USA) for plotting maps. The background was subtracted by automatically applied the MatLab function (MSBACKADJ, stepsize 50) to each individual spectrum. Cosmic rays on individual CCD pixels were removed by home-made MatLab codes. Peak heights were determined by means of the automatic peak finder (maximums in the selected area) based on MatLab, which was used for intensity analysis. For a selected peak, the noise level was calculated by Matlab using a root mean square (RMS) function in the nearby area, and the individual threshold (signal-to-noise ratio over three) was calculated for each spectrum (after aforementioned preprocessing) at every pixel of the maps. In the peak ratio maps, the intensity of individual peaks would be set as zero when the value of their signal-to-noise ratio was less than three, otherwise set as the maximum. For all individual TER spectra extracted from imaging and DFT calculated spectra, the data were processed in Origin (9.0, OriginLab, USA) to normalize and fit with a Gaussian function.

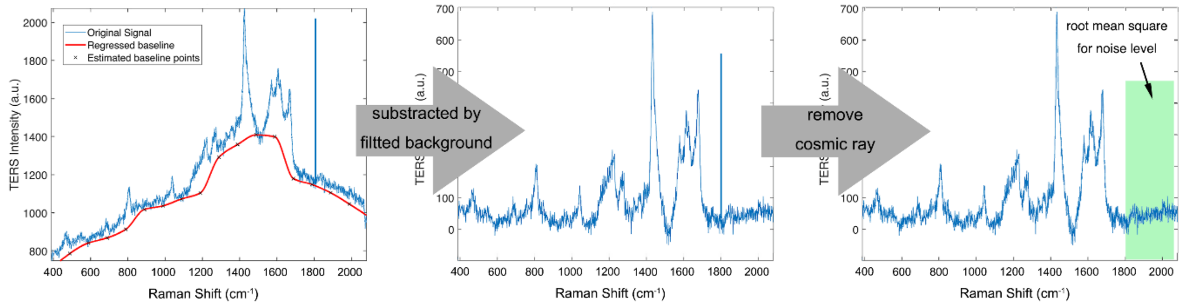

Supplementary Figure 34 | Data batch processing of a typical TERS spectrum in the TERS maps for intensity and peak ratio analysis.[37, 38]

In order to evaluate the LSP contribution in the (semi-)quantitative spectral analysis, we also followed a reported method (by Ren and co-workers) to correct the influence of the plasmon resonance dispersion on the relative intensity of the TERS peaks.[39] Interestingly, the LSP shape (background (Bg)) is almost negligible for the given data set/analysis with and without the background division (Supplementary Figure P2). However, some variations in relative band intensities (e.g., 1566, 1600, and 1670 cm^{-1}) can be found when applying the two processing methods (Supplementary Figs. P1, 2). That is mainly due to the difference in defining and fitting the background baselines (MSBACKADJ *vs.* ALSS).

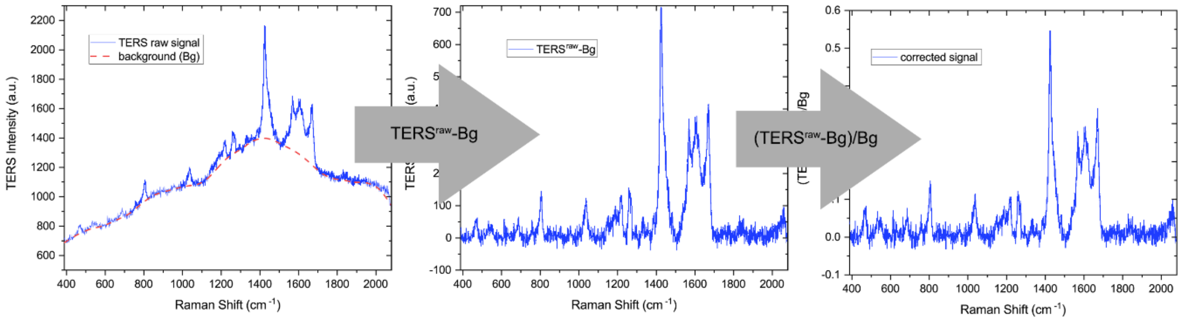

Supplementary Figure 35 | Data processing of a typical TERS spectrum with and without the background division. The background baseline is set and fitted by the asymmetric least squares smoothing (ALSS) function in Origin 9.0 (OriginLab, USA).

4. Supplementary References

1. Wang W, Shao F, Kröger M, Zenobi R, Schlüter AD. Structure Elucidation of 2D Polymer Monolayers Based on Crystallization Estimates Derived from Tip-Enhanced Raman Spectroscopy (TERS) Polymerization Conversion Data. *J. Am. Chem. Soc.* **141**, 9867-9871 (2019).

2. Müller V*, et al.* Structural Characterization of a Covalent Monolayer Sheet Obtained by Two-Dimensional Polymerization at an Air/Water Interface. *Angew. Chem. Int. Ed.* **56**, 15262-15266 (2017).

3. Vadai M, Angell DK, Hayee F, Sytwu K, Dionne JA. In-situ observation of plasmon-controlled photocatalytic dehydrogenation of individual palladium nanoparticles. *Nat. Commun.* **9**, 4658 (2018).

4. Zhou L*, et al.* Quantifying hot carrier and thermal contributions in plasmonic photocatalysis. *Science* **362**, 69 (2018).

5. Jiang R, Li B, Fang C, Wang J. Metal/Semiconductor Hybrid Nanostructures for Plasmon-Enhanced Applications. *Adv. Mater.* **26**, 5274-5309 (2014).

6. Zhan C, Chen X-J, Yi J, Li J-F, Wu D-Y, Tian Z-Q. From plasmon-enhanced molecular spectroscopy to plasmon-mediated chemical reactions. *Nat. Rev. Chem.* **2**, 216-230 (2018).

7. Shao F, Zenobi R. Tip-enhanced Raman spectroscopy: principles, practice, and applications to nanospectroscopic imaging of 2D materials. *Anal. Bioanal. Chem.* **411**, 37-61 (2019).

8. Gellé A, Jin T, de la Garza L, Price GD, Besteiro LV, Moores A. Applications of Plasmon-Enhanced Nanocatalysis to Organic Transformations. *Chem. Rev.* **120**, 986-1041 (2020).

9. Szczerbiński J, Gyr L, Kaeslin J, Zenobi R. Plasmon-Driven Photocatalysis Leads to Products Known from E-beam and X-ray-Induced Surface Chemistry. *Nano Lett.* **18**, 6740-6749 (2018).

10. Schlüter AD, Weber T, Hofer G. How to use X-ray diffraction to elucidate 2D polymerization propagation in single crystals. *Chem. Soc. Rev.* **49**, 5140-5158 (2020).

11. Müller V*, et al.* A Two-Dimensional Polymer Synthesized at the Air/Water Interface. *Angew. Chem. Int. Ed.* **57**, 10584-10588 (2018).

12. Murray DJ*, et al.* Large Area Synthesis of a Nanoporous Two-Dimensional Polymer at the Air/Water Interface. *J. Am. Chem. Soc.* **137**, 3450-3453 (2015).

13. Ozbay E. Plasmonics: merging photonics and electronics at nanoscale dimensions. *Science* **311**, 189-193 (2006).

14. Wouters D, Schubert US. Nanolithography and Nanochemistry: Probe-Related Patterning Techniques and Chemical Modification for Nanometer-Sized Devices. *Angew. Chem. Int. Ed.* **43**, 2480-2495 (2004).

15. Lange RZ, Hofer G, Weber T, Schlüter AD. A Two-Dimensional Polymer Synthesized through Topochemical [2 + 2]-Cycloaddition on the Multigram Scale. *J. Am. Chem. Soc.* **139**, 2053-2059 (2017).

16. Ding S-Y, You E-M, Yi J, Li J-F, Tian Z-Q. Further expanding versatility of surface-enhanced Raman spectroscopy: from non-traditional SERS-active to SERS-inactive substrates and single shell-isolated nanoparticle. *Faraday Discuss.* **205**, 457-468 (2017).

17. Zheng L-Q, Servalli M, Schlüter AD, Zenobi R. Tip-enhanced Raman spectroscopy for structural analysis of two-dimensional covalent monolayers synthesized on water and on Au (111). *Chem. Sci.* **10**, 9673-9678 (2019).

18. Zhang Q, Wang H. Facet-Dependent Catalytic Activities of Au Nanoparticles Enclosed by High-Index Facets. *ACS Catal.* **4**, 4027-4033 (2014).

19. Van Damme J, Du Prez F. Anthracene-containing polymers toward high-end applications. *Prog. Polym. Sci.* **82**, 92-119 (2018).

20. Ihmels H, Luo J. The reversible [4+4] photocycloaddition of acridizinium derivatives. *J Photochem. Photobiol. A* **200**, 3-9 (2008).

21. Wang W, Schlüter AD. Synthetic 2D Polymers: A Critical Perspective and a Look into the Future. *Macromol. Rapid Commun.* **40**, 1800719 (2019).

22. You C-Y*, et al.* Plasmon-Enhanced Fluorescence of Phosphors Using Shell-Isolated Nanoparticles for Display Technologies. *ACS Appl. Nano Mater.* **3**, 5846-5854 (2020).

23. Li C-Y*, et al.* "Smart" Ag Nanostructures for Plasmon-Enhanced Spectroscopies. *J. Am. Chem. Soc.* **137**, 13784-13787 (2015).

24. Johnson PB, Christy RW. Optical Constants of the Noble Metals. *Phys. Rev. B* **6**, 4370-4379 (1972).

25. Yang Z, Aizpurua J, Xu H. Electromagnetic field enhancement in TERS configurations. *J. Raman Spectrosc.* **40**, 1343-1348 (2009).

26. Chen Y*, et al.* Broadband unidirectional scattering in visible ranges and controllable hot-spot spatial transfer via a single nanoparticle. *Appl. Surf. Sci.* **528**, 146489 (2020).

27. Jiang S*, et al.* Distinguishing adjacent molecules on a surface using plasmon-enhanced Raman scattering. *Nat. Nanotechnol.* **10**, 865-869 (2015).

28. Zhang R*, et al.* Chemical mapping of a single molecule by plasmon-enhanced Raman scattering. *Nature* **498**, 82-86 (2013).

29. Stadler J, Oswald B, Schmid T, Zenobi R. Characterizing unusual metal substrates for gap-mode tip-enhanced Raman spectroscopy. *J. Raman Spectrosc.* **44**, 227-233 (2013).

30. Hutter J, Iannuzzi M, Schiffmann F, VandeVondele J. cp2k: atomistic simulations of condensed matter systems. *WIRES Comput. Mol. Sci.* **4**, 15-25 (2014).

31. Perdew JP, Burke K, Ernzerhof M. Generalized Gradient Approximation Made Simple. *Phys. Rev. Lett.* **77**, 3865-3868 (1996).

32. Grimme S. Semiempirical GGA-type density functional constructed with a long-range dispersion correction. *J. Comput. Chem.* **27**, 1787-1799 (2006).

33. VandeVondele J, Hutter J. Gaussian basis sets for accurate calculations on molecular systems in gas and condensed phases. *J. Chem. Phys.* **127**, 114105 (2007).

34. VandeVondele J, Krack M, Mohamed F, Parrinello M, Chassaing T, Hutter J. Quickstep: Fast and accurate density functional calculations using a mixed Gaussian and plane waves approach. *Comput. Phys. Commun.* **167**, 103-128 (2005).

35. Goedecker S, Teter M, Hutter J. Separable dual-space Gaussian pseudopotentials. *Phys. Rev. B* **54**, 1703 (1996).

36. Hartwigsen C, Gœdecker S, Hutter J. Relativistic separable dual-space Gaussian pseudopotentials from H to Rn. *Phys. Rev. B* **58**, 3641 (1998).

37. Shao F, Dai W, Zhang Y, Zhang W, Schlüter AD, Zenobi R. Chemical Mapping of Nanodefects within 2D Covalent Monolayers by Tip-Enhanced Raman Spectroscopy. *ACS Nano* **12**, 5021-5029 (2018).

38. Shao F, Müller V, Zhang Y, Schlüter AD, Zenobi R. Nanoscale Chemical Imaging of Interfacial Monolayers by Tip-Enhanced Raman Spectroscopy. *Angew. Chem. Int. Ed.* **56**, 9361-9366 (2017).

39. Lin K-Q*, et al.* Plasmonic photoluminescence for recovering native chemical information from surface-enhanced Raman scattering. *Nat. Commun.* **8**, 14891 (2017).
